# Prediction of monthly dry days with machine learning algorithms: a case study in Northern Bangladesh

**DOI:** 10.1038/s41598-022-23436-x

**Published:** 2022-11-16

**Authors:** Shabbir Ahmed Osmani, Jong-Suk Kim, Changhyun Jun, Md. Wahiduzzaman Sumon, Jongjin Baik, Jinwook Lee

**Affiliations:** 1 Department of Smart Cities, Chung-Ang University, Seoul, Republic of Korea; 2 State Key Laboratory of Water Resources and Hydropower Engineering Science, Wuhan University, Wuhan, 430072 People’s Republic of China; 3 Department of Civil and Environmental Engineering, Chung-Ang University, Seoul, Republic of Korea; 4 Department of Civil Engineering, Leading University, Sylhet, Bangladesh

**Keywords:** Climate sciences, Projection and prediction

## Abstract

Dry days at varied scale are an important topic in climate discussions. Prolonged dry days define a dry period. Dry days with a specific rainfall threshold may visualize a climate scenario of a locality. The variation of monthly dry days from station to station could be correlated with several climatic factors. This study suggests a novel approach for predicting monthly dry days (MDD) of six target stations using different machine learning (ML) algorithms in Bangladesh. Several rainfall thresholds were used to prepare the datasets of monthly dry days (MDD) and monthly wet days (MWD). A group of ML algorithms, like Bagged Trees (BT), Exponential Gaussian Process Regression (EGPR), Matern Gaussian Process Regression (MGPR), Linear Support Vector Machine (LSVM), Fine Trees (FT) and Linear Regression (LR) were evaluated on building a competitive prediction model of MDD. In validation of the study, EGPR-based models were able to better capture the monthly dry days (MDD) over Bangladesh compared to those by MGPR, LSVM, BT, LR and FT-based models. When MDD were the predictors for all six target stations, EGPR produced highest mean *R*^2^ of 0.91 (min. 0.89 and max. 0.92) with a least mean *RMSE* of 2.14 (min. 1.78 and max. 2.69) compared to other models. An explicit evaluation of the ML algorithms using one-year lead time approach demonstrated that BT and EGPR were the most result-oriented algorithms (*R*^2^ = 0.78 for both models). However, having a least *RMSE,* EGPR was chosen as the best model in one year lead time. The dataset of monthly dry–wet days was the best predictor in the lead-time approach. In addition, sensitivity analysis demonstrated sensitivity of each station on the prediction of MDD of target stations. Monte Carlo simulation was introduced to assess the robustness of the developed models. EGPR model declared its robustness up to certain limit of randomness on the testing data. The output of this study can be referred to the agricultural sector to mitigate the impacts of dry spells on agriculture.

## Introduction

The global temperature increased by 0.6 °C (0.4–0.8 °C) from 1901 to 2001, highlighting the warming of the Earth in recent decades^1^. The resulting extreme temperatures, precipitation, and continuous wet or dry conditions have severely impacted human activities and the ecosystem^2–4^. Similarly, droughts due to extreme temperatures and dry conditions have become increasingly commonplace worldwide^5,6^. These drought events and their frequency are directly affected by global warming, with 30% of the Earth’s surface expected to experience as much as twice the drought intensity by the end of this century, affecting most of the global population^5–7^. Hence, the occurrence of droughts is a prime area of focus for monitoring and management from agricultural point of view to ensure food security in affected areas.

Bangladesh is characterized as one of the most environmentally vulnerable countries in the world^8–10^ owing to the substantial adverse impacts of climate change, in combination with its geographical location and socio-economic conditions. Bangladesh is less adaptable to sustain adverse effects of climate change because of its developing economy, geography, and high population density, which lead to a low adaptive capacity^11^. The adverse impacts of climate change are generally visible in the agricultural sector, as most agricultural processes depend on rainfall^12^. Agriculture contributes approximately 14% to Bangladesh’s GDP and employs approximately 40% of its labor force^13^. As a result of reduced or no rainfall, regional droughts currently affect approximately 2.5 million and 1.2 million ha of agricultural land in a year in the wet and dry seasons, respectively^14^. Therefore, the prediction of dry days could be an approach for applying measures to mitigate the regional effects of prolonged dry spells.

Droughts have been identified and characterized at different scales. There are four types of droughts^15^: meteorological, agricultural, hydrological, and socio-economic. Meteorological droughts are defined based on the degree of dryness (an expression of precipitation departure) and the duration of the dry period^15–19^. Agricultural drought occurs when there is insufficient soil moisture to meet the needs of a particular crop in a specific time owing to deficient precipitation for an extended period. Hydrological drought occurs when there are deficiencies in surface and subsurface water supplies, based on measurements of streamflow and lake, reservoir, and groundwater levels. Meanwhile, socioeconomic drought can be referred to the situations when the supplied volume of water is less than the demand of water in a specific region^20^. Hoyt^21^ defined socioeconomic drought as occurring insufficient precipitation to meet the needs of human activities. This concept was expanded by Hoyt^22^ in 1942 by stating that socio-economic development in a region demands more water than normally available.

Multiple drought indices (DIs) have been used to define drought events and their intensities^23^ to identify the spatiotemporal distribution of droughts^24^. The standardized precipitation index (SPI)^25^ is the most popular meteorological drought index, based on monthly precipitation^26^. The effective drought index (EDI)^27^ is another useful tool for distinguishing the characteristics of droughts. However, the application of SPI found some limitations in defining short and long-term droughts where EDI showed its effectiveness on detecting long and short-term droughts^26,28^. In addition, different monthly SPIs are found in a particular month, while EDI provides a single value, which causes misinterpretation of droughts for that month. Other studies^28–30^ have found that EDI can detect a high range of drought events. Moreover, precipitation and temperature define another drought index named as Standardized Precipitation Evapotranspiration Index (SPEI)^31^. The superiority of SPEI focuses by combining the effects of temperature variability on drought assessments.

Beside the drought indices, some other ways were also followed to characterize a dry event or period. A dry period was referred with prolonged consecutive dry days with little or no precipitation over a specific duration^32–35^. Some meteorologists and climatologists designated a dry spell with precipitation less than 2 or 5 mm^27^. Drought events were characterized by 15 consecutive dry days^35,36^ or a long dry period with 25 days consecutive dry days^35^. Moreover, climate scenarios were effectively presented through wet and dry periods^37–44^ and argued that wet and dry periods are useful indicators of weather^45,46^. In Switzerland, wet and dry periods were found capable to extrapolate the climate through spatial and temporal trends of wet and dry periods^38^. Dry days were found generating heat wave and in tropical, weather dry days were directly or indirectly related to heatwave. Heatwave vulnerability was used to identify the hot zones in a locality^47^ through climatic, socio-economic, physiological, and environmental parameters. Heat wave was also analyzed by the effect of the North Atlantic Oscillation^48^. Similarly, in both day and nighttime situations, a dense meteorological network was used to study urban and rural air temperatures where the urban heat index (UHI) was the highest when weather was dry^49^. Hence, dry days have logical relations on producing heatwaves.

There were a limited number of researches on predicting future dry days, based on monthly cumulative dry days. Other researchers, for example, mainly focused on Monthly Consecutive Dry Days (MCDD) over Japan^50^ to present zonal climate and established the application of consecutive dry days. Meanwhile, a study^51^ on monthly dry days (MDD) argued that MDD cannot be a direct description of defining a particular type of drought, but it would be meaningful to find trends of changes of dry spells in different months. This study was motivated to establish some new approaches on finding correlations of MDD and monthly wet days (MWD) in between stations.

Dry period or drought prediction and forecasts can be performed using either physical or data-driven models. A flood forecasting data-driven model^52^ showed data-driven models require minimal information for a short duration to build a result oriented model. Precipitation and droughts were also forecasted using statistical data driven models in several studies. For example, linear regression^53^, support vector machine (SVM)^54^ and artificial neural network (ANN)^55^ were extensively used for long term drought prediction using SPI. These data-driven models took rainfall or drought relevant variables in the previous months as inputs, and the rainfall or drought indicators as outputs. ANN based models were more capable for forecasting droughts compared to others. Furthermore, ANN provided greater performance than multiple linear regression in forecasting SPEI in Wilsons Promontory in Australia^56^. Several ML algorithms were also implemented on rainfall forecasting^57^ and the results were consistently better using auto correlation functions.

However, in Pakistan, the prediction of SPEI showed the superiority of SVM over ANN and k-nearest neighbor (KNN)^58^. Another study^59^ established the accuracy of SVM over ANN on predicting SPI over Iran. The studies were accomplished with the fact that ML models have higher advantage on producing better accuracy by utilizing only hydro-meteorological data rather than considering the inherent physical processes^60^.

Drought forecasting with longer lead times and higher accuracy is of significant value in agriculture applications. A study on different lead times phenomena among different drought studies admitted the challenges on lead time forecasting^61^. Among different ML algorithms, artificial neural network (ANN) based models were used in several studies and proved its effectiveness on forecasting droughts from 1 to 12 months lead time^62–64^.

Uncertainty analysis on a proposed model confirms the robustness of the model. This uncertainty could be originated from a systematic error or by a random error. Uncertainty of different hydrological models on predicting climate events has been established as a vital approach to quantify the domain of study inputs or model parameters. In these studies, Monte-Carlo sampling-based methods were adopted^65–67^. Different ranges of random data from the input parameters were generated to see the effect on the original level of output. For example, Monte Carlo simulation was used to perform uncertainty in different water model parameters^68,69^ and checked the robustness of the proposed models.

This study was intended to deal with monthly dry days (MDD) and monthly wet days (MWD) instead of consecutive dry days. And finding regressions among MDD and MWD would claim the novelty of the study. It is not to visualize any dry spell or dry period in the study area. Rather, finding a strong regression among MDD of different climate stations through several machine learning algorithms was initiated. Here, a dry day was defined when a day has a rainfall less than 2 mm instead of 1 mm^50^ and MDD was the cumulative dry days in every month. Datasets of monthly wet days, defined by several daily rainfall thresholds, were also used to establish regressions with MDDs. Different ML algorithms, like Fine Tree (FT), Bagged Trees (BT), Linear Regression (LR), Linear Support Vector Machine (LSVM), Exponential GPR (EGPR) and Matern GPR (MGPR) were incorporated to find a strong prediction model of MDD of the climate stations. The outcome of the study was also assessed its robustness using Monte Carlo simulation with different ranges of random datasets.

## Results

### Statistical summary

**Figure 1:**
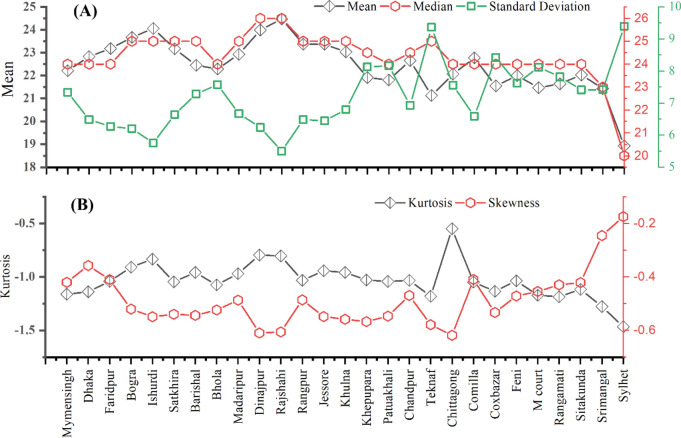
Descriptive statistics of MDD of all stations in this study.

MDD of 27 stations have varied statistical responses. Figure 1A represents diversified ranges of mean, median and standard deviation. Several stations have high and low reaches in mean, median and standard deviation. The datasets are normally distributed since mean and median are very close to each other. Negative skewness depicts a higher concentration of data to the right. Skewness values in the range of − 2 to + 2 are generally acceptable^70^. The datasets are found to be less skewed as the skewness was in the range of − 0.6 to − 0.2. It means the datasets are very close to normally distributed.

In contrast, Kurtosis defines the relative peaked-ness or flatness of the data relative to normal distribution. Figure 1B clearly depicts all negative values within − 1.5 to − 0.5 which means mean thinner tails. Kurtosis value in the range of − 2 to + 2 is generally acceptable to prove normal univariate distribution^70^.

### Prediction of MDD

The performance of the ML models for the prediction of MDD was determined and assessed using multiple approaches. In the first approach, only MDD of all stations were considered as study dataset. Every target station was taken as response while remaining 26 stations were the predictors. In the second approach, MWDs of all 26 stations (other than the target) were used as predictors. In the third approach, integrated monthly dry and wet days (MDWDs) at all stations were utilized as predictors. From the dataset of 35 years, 23 years (2/3rd) of data were used for training and 12 years (1/3rd) of data were used for testing. Two performance indicators, *R*^2^ and *RMSE*, of each developed model stratified the efficiency on prediction strategy.

Out of all, EGPR and MGPR secured better results than any other algorithm in training dataset (Table 1). More particularly, EGPR routinely outperformed all other algorithms, with the highest mean R^2^ (~ 1.00) for the first and third approaches. MGPR, on the other hand, for the same first and third approaches, has the second-best R^2^ (~ 0.99). Reasonably, performance levels of the developed models are a bit deviated for the testing period.

**Table 1:**
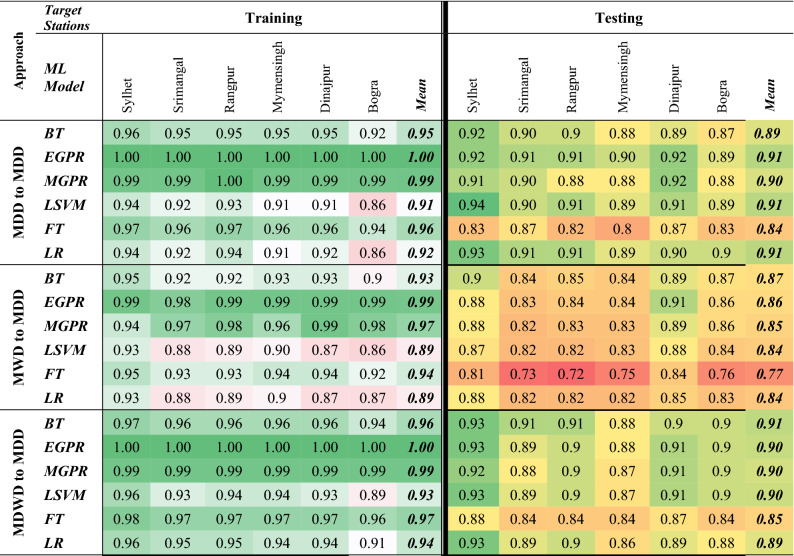
Values of R^2^ from the ML models for the approaches (1) MDD to MDD (2) MWD to MDD & (3) MDWD to MDD.

Significant values are in bold and italics.

Focusing at the testing results, through the second approach, BT outraced the performance of other algorithms. The lowest average score of *R*^*2*^ (~ 0.77) was produced by FT. All other responses using the second approach had a non-significant *R*^2^ of 0.87 by BT. But for the first approach, EGPR, LSVM and LR, each algorithm scored a mean *R*^2^ of 0.91 while they scored *RMSE* of 2.14, 2.16 and 2.16, respectively. In contrast, using the third approach, EGPR, MGPR, and LSVM, each have a bit reduced mean *R*^2^ (0.90) and higher *RMSE* of 2.19, 2.26 and 2.21, respectively. Therefore, EGPR has the optimum scores of *R*^*2*^ and *RMSE* by using the data of the second approach.

On the other hand, while prediction of MDD was tested from MDWD using the third approach, BT scored a highest mean *R*^2^ (0.91) and second lowest mean *RMSE* of 2.20 (Table 2). In summary, comparing all scores, EGPR has the lowest mean *RMSE* of 2.14 with highest *R*^2^ of 0.91, Hence, the study found EGPR as the best model and the 1st approach was identified as the best approach.

**Table 2:**
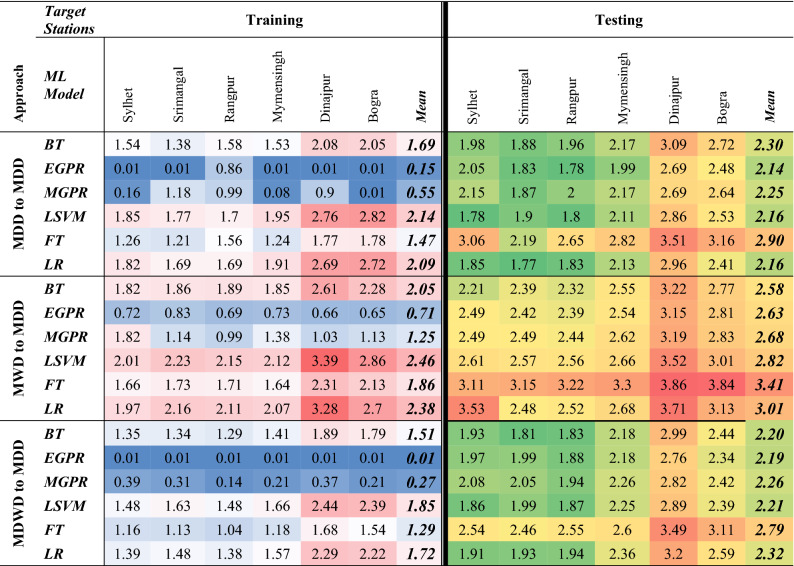
RMSE of the ML models for the approaches (1) MDD to MDD (2) MWD to MDD & (3) MDWD to MDD.

Significant values are in bold and italics.

**Figure 2:**
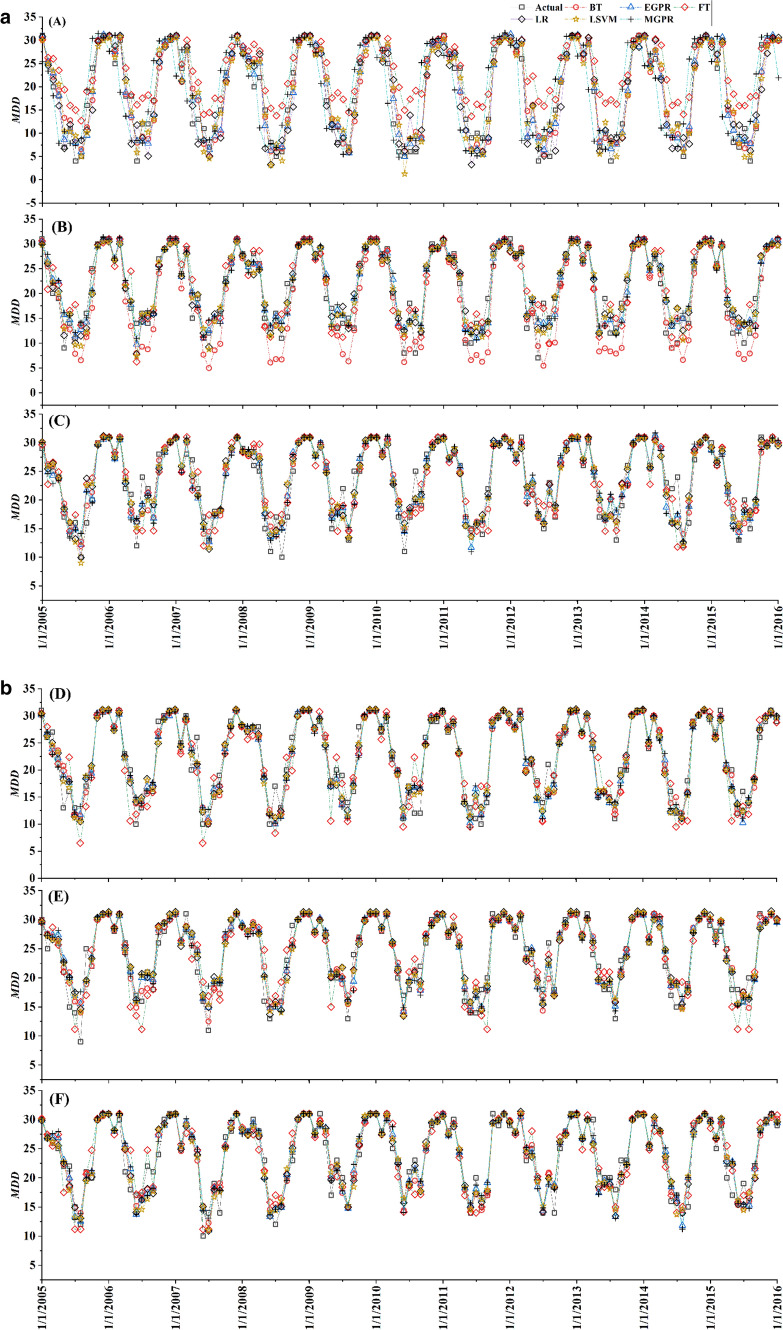
(**a**) Actual and predicted MDD using all ML models when targets are: A = Sylhet, B = Srimangal, C = Rangpur. (**b**) Actual and predicted MDD using all ML models when targets are: D = Mymensingh, E = Dinajpur and F = Bogra.

Figure 2a and b represented a comparison of the predicted MDD developed by all ML models for the six target stations following the first approach. The predicted values of Sylhet are traced well by LSVM rather than any other model where EGPR and LR picked the most of the actual values of MDD of Srimangal. Meanwhile, Rangpur station was caught by EGPR, LSVM and LR for better accuracy whilst EGPR and MGPR worked well for prediction of Dinajpur. Therefore, individual model goes fit for the individual station while combined performance considering least *RMSE* suggest EGPR as the best algorithm.

### Lead time forecasting

The key objective of lead time approach was to evaluate the effectiveness of ML techniques for developing a reliable forecasting model that can be used to manage dry periods in advance by the agricultural industry, and the authority could take necessary precautions against possible dry spells. One year lead time was considered to step up the scenario of dry days in one ahead. All the three identical approaches and their predictors were employed to identify the most significant input datasets building a MDD forecasting model with high *R*^2^ with low *RMSE*. The training dataset contained predictors from 1982 to 2003 and responses from 1983 to 2004. The testing period for the predictors was from 2004 to 2016, and consequently, the forecasted period was 2005–2017.

The results of the lead time approach in Table 3 showed a consistent regression for having better forecasting on MDD. In comparison, BT and EGPR models, for the third approach, produced highest *R*^*2*^ and least *RMSE* compared to other models. Having an identical mean *R*^2^ of 0.78, BT and EGPR are the stronger models in this simulation for predicting MDD with one year lead. However, the performance of EGPR outraced BT on the basis of less *RMSE*.

**Table 3:**
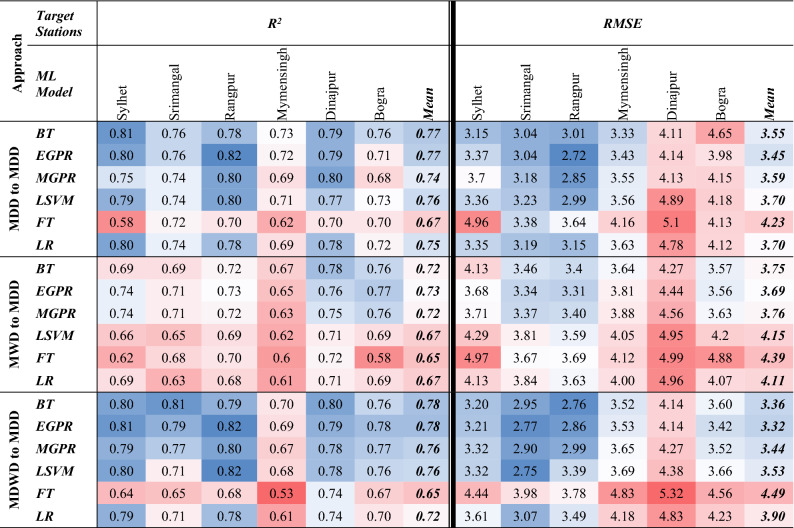
R^2^ & RMSE of the ML models for the approaches (1) MDD to MDD (2) MWD to MDD & (3) MDWD to MDD using testing dataset.

Significant values are in bold and italics.

The performance of LSVM was not satisfactory for having a low *R*^2^ (0.71) even though it had the lowest *RMSE* (2.75) for forecasting Srimangal. In addition, FT produced highest *RMSE* (5.32) for Dinajpur and minimum *R*^2^ (0.53) for Mymensingh. And, EGPR and LSVM were competitive for Rangpur having highest *R*^*2*^ with varied *RMSE.* Every ML algorithm uses specific set of model parameters and coefficients to generate prediction models using variety of input datasets with minimized prediction errors by using different performance indicators like *RMSE* and *R*^2^ values^57,71^. Likely, performance levels are fluctuated here for different ML algorithms as well as input datasets.

**Figure 3:**
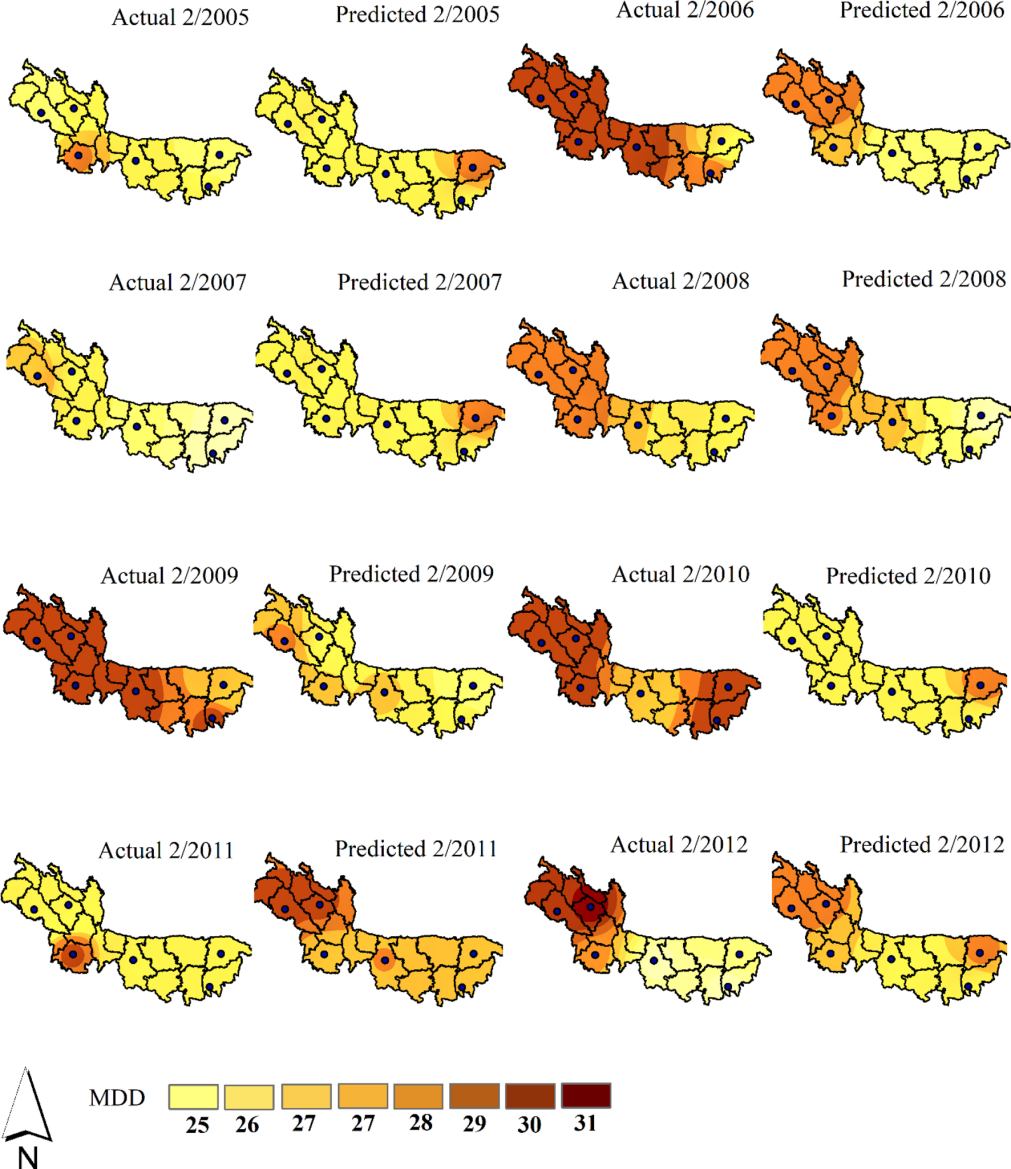
Spatiotemporal distribution of actual and predicted MDD using EGPR at all stations.

**Figure 4:**
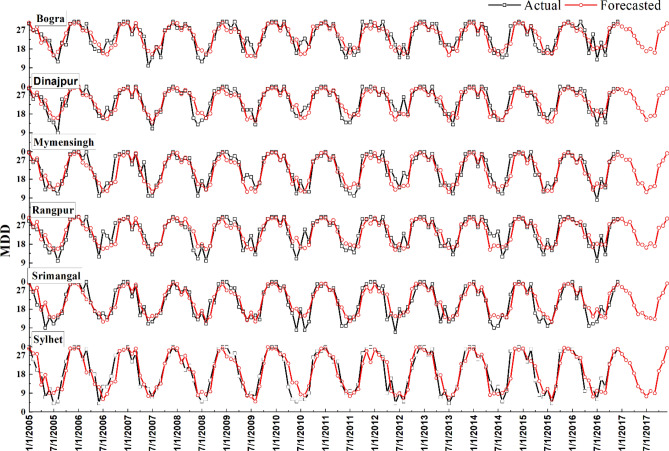
Forecasted MDD using EGPR for Sylhet, Srimangal, Rangpur, Mymensingh, Dinajpur and Bogra stations.

The results of the testing dataset using EGPR are extrapolated through Figs. 3 and 4. Most of the highs and lows are easily captured by the model. However, some points of MDD have a bit fluctuation. For example, year 2006 has significant deviation of predicted values with the actuals. But these are very little compared to the true patterns of prediction. Particularly, Sylhet and Bogra have a very good one-year lead time prediction throughout the testing period.

### Sensitivity analysis

Sensitivity analysis finds the efficiency of input parameters in developing data driven models. The focus is centered on the behavior of input parameters on the variation of the model output. In fact, different parameters have different (sometimes extreme) effect on the model’s outcome. Given that some parameters play significant roles, while others are marginally important, make sensitivity analysis a valuable tool.

**Figure 5:**
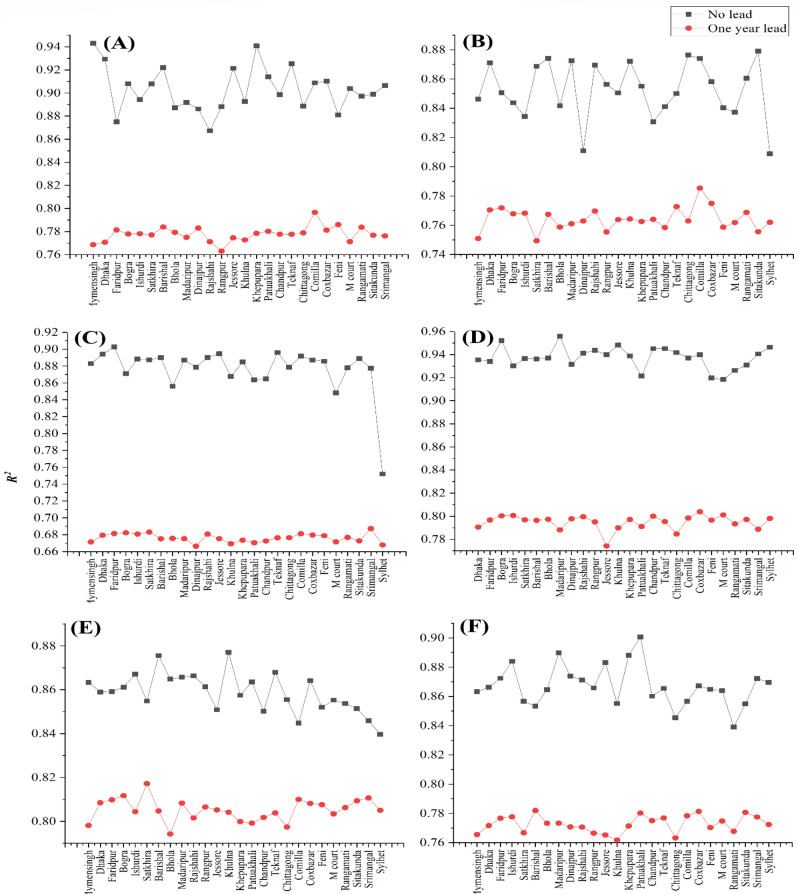
Sensitivity of different stations for predicting MDD of six target stations: A = Sylhet, B = Srimangal, C = Rangpur, D = Mymensingh, E = Dinajpur and F = Bogra.

To perform sensitivity analysis, a scenario was assumed that a station did not have any study data in the testing period. Keeping every station of Northern Bangladesh as target, all the 26 stations were checked through the developed EGPR model. Figure 5 summarizes the output levels of prediction for the six target stations. Significance of the station parameters in model validation is usually checked through this process. Results showed variety of significant stations to reach to the desired levels of prediction.

Rangpur is most sensitive when predicting MDD of Sylhet for one year lead time where Faridpur and Rajshahi were sensitive without any lead time (Fig. 5). Again, Mymensingh and Khepupara are found least sensitive without considering any lead time while Comilla was the least sensitive with one year lead targeting Sylhet.

Sylhet is significant for targeting Srimangal, Rangpur and Dinajpur while there is no any significant station predicting Mymensingh for zero lead time. In summary, for different target station with no lead time, the *R*^2^ values of predicted models lie within 0.90 ± 0.04 for Sylhet, 0.84 ± 0.03 for Srimangal, 0.88 ± 0.02 for Rangpur, 0.94 ± 0.02 for Mymensingh, 0.86 ± 0.02 for Dinajpur, and 0.87 ± 0.03 for Bogra. In contrast, when considering one year lead time, *R*^2^ values remain around 0.78 for targeting Sylhet, Srimangal, Mymensingh and Bogra where *R*^2^ was approximately 0.68 and 0.81 for Rangpur and Dinajpur respectively.

In summary of the sensitivity analysis, it is concluded that a particular station was not highly sensitive for most of the target stations. Specifically, Sylhet and Dinajpur were found sensitive solely for Rangpur and Srimangal stations, respectively. Hence, sensitivity analysis for this intended procedure and models of the study is less result oriented.

### Uncertainty analysis

An uncertainty analysis shows the propagation of uncertainty through the hydrological models and to derive meaningful uncertainty bounds of the model simulations^72^. This study incorporated two scenarios to perform uncertainty analysis. At first, any station was assumed to have random data within different coefficient of variations (*CV*). Secondly, any two stations were random within different *CVs*. Here, 0.01, 0.05, 0.1, 0.5, 1 and 2 are the *CVs* had been considered to do the simulation.

**Figure 6:**
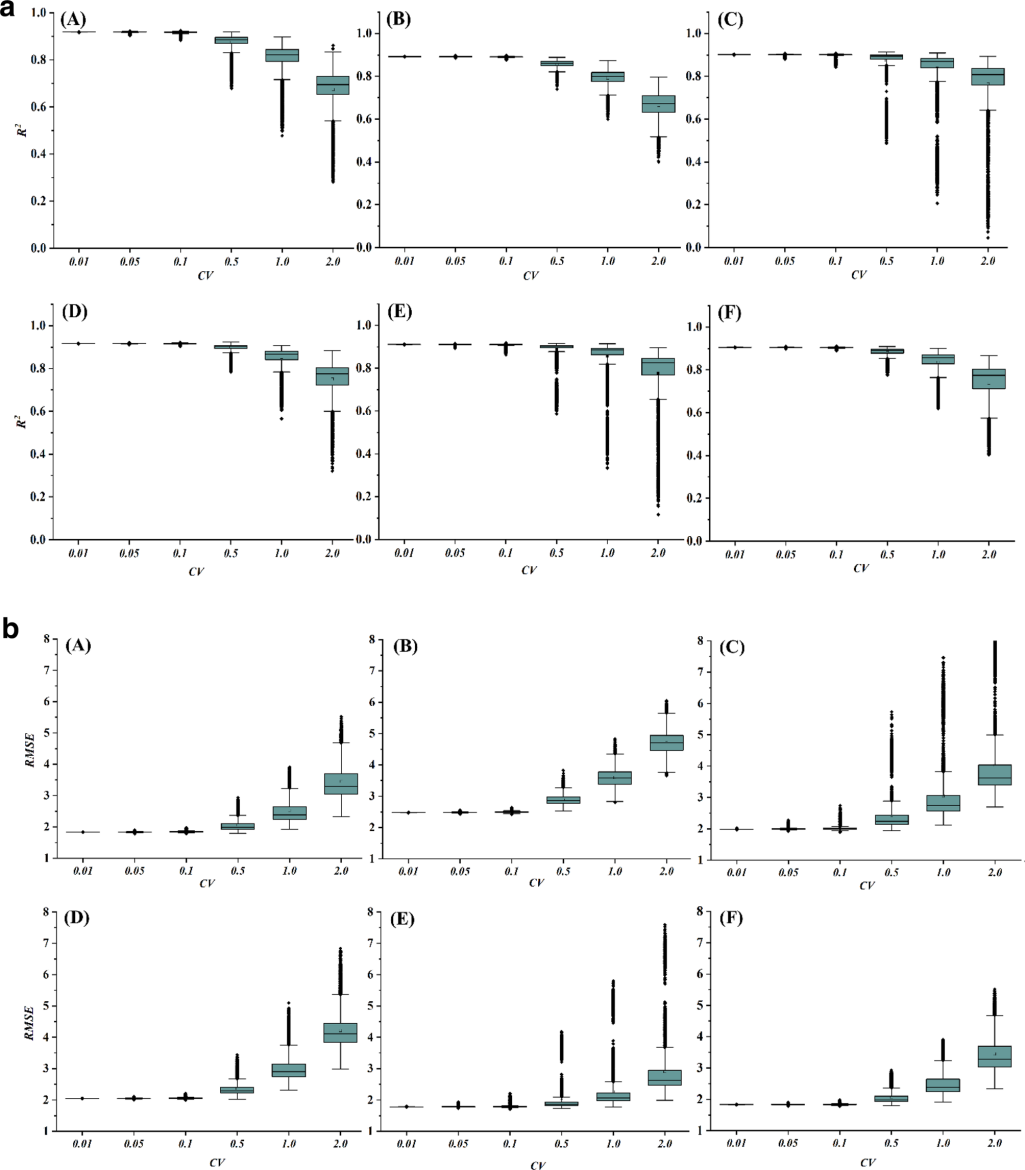
(**a**) Variation of *R*^*2*^ when a station has random data with different *CV*: A = Sylhet, B = Srimangal, C = Rangpur, D = Mymensingh, E = Dinajpur and F = Bogra. (**b**) Variation of *RMSE* values when a station has random data with different *CV*: A = Sylhet, B = Srimangal, C = Rangpur, D = Mymensingh, E = Dinajpur and F = Bogra.

**Figure 7:**
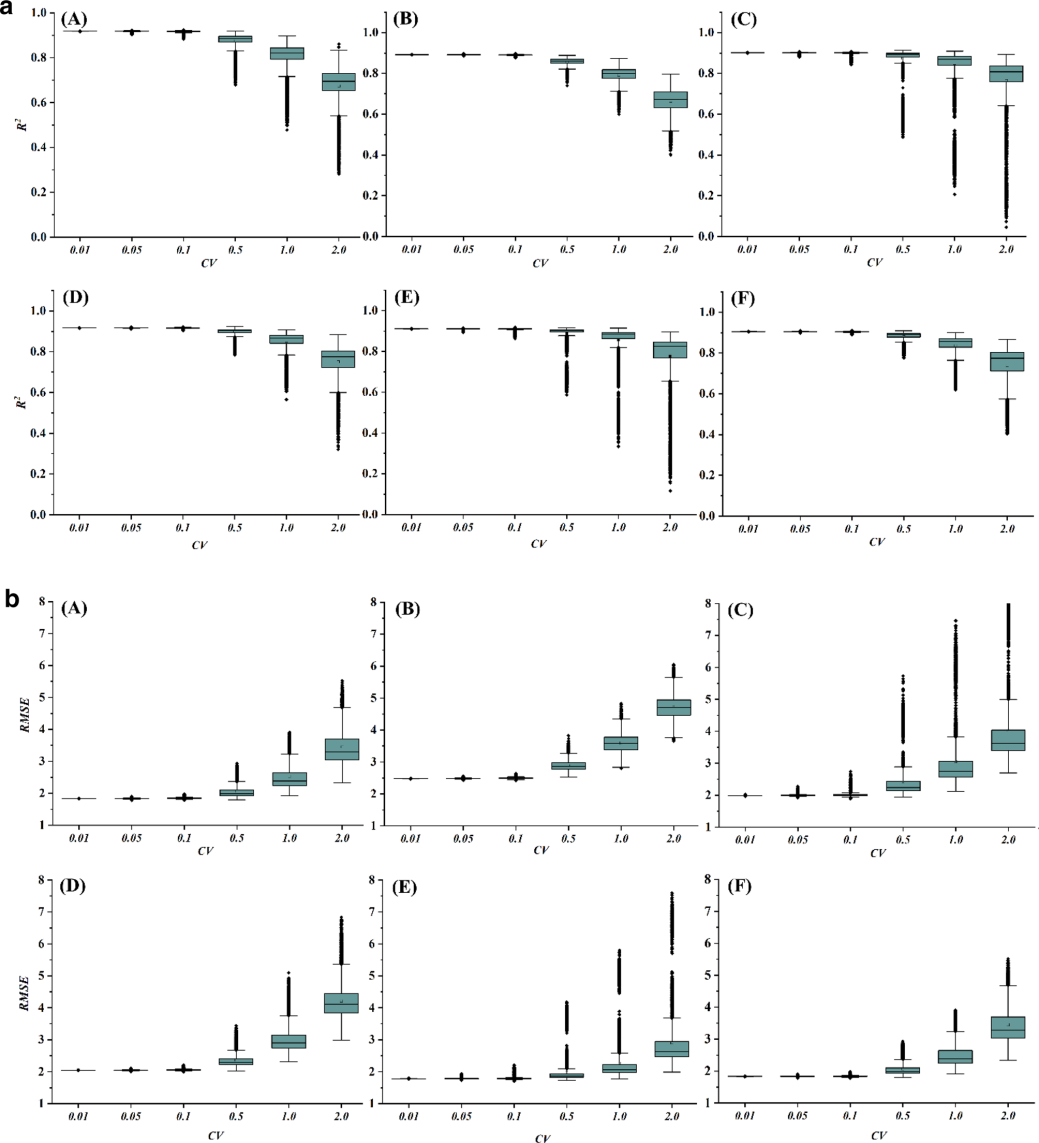
(**a**) Variation of *R*^*2*^ values when two stations have random data with different *CV*: A = Sylhet, B = Srimangal, C = Rangpur, D = Mymensingh, E = Dinajpur and F = Bogra. (**b**) Variation of *RMSE* values when two stations have random data with different *CV*: A = Sylhet, B = Srimangal, C = Rangpur, D = Mymensingh, E = Dinajpur and F = Bogra.

The typical syntax to generate random data is:
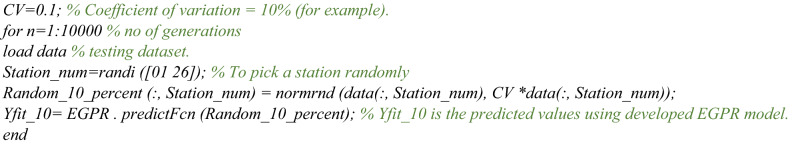


To comply with Monte Carlo simulations, total 10,000 sets^73^ of new datasets were generated for a particular *CV*. When Sylhet was the target, for example, a station was picked randomly among the 26 stations and data of testing period of that station was generated randomly with a specific *CV*. This was repeated for 10,000 times for that *CV*. Every dataset was then evaluated by the developed EGPR model. The statistical details of the results are summarized through the boxplots in Figs. 6 and 7.

#### Case A: a single station was random

This type of uncertainty would be originated due to the errors in data recording, data processing, or errors in systems. The results of the analyses through Fig. 6a and b ensure that the models are consistent for the randomness of the predictors up to the *CV* of 0.1. If any station data vary at *CV* of 0.5 or more, the performance of the models are getting deviated.

#### Case B: any two stations were random

If a situation arises when any two stations are having random data with different spikes then the developed should also work with the new testing dataset. Figure 7a and b represent the outputs of this scenario. The analysis of this type of randomness produced quite similar responses compared to the randomness of one station. However, for Ranpur, Mymensingh, Dinajpur and Bogra, the robustness of the EPGR was extended up to the random data with *CV* of 0.5.

## Discussion

The data analysis and the developed models through different sets of inputs and outputs represented a detail data driven model for forecasting a climate parameter. Simulation in this study generated some key outputs for the prediction of MDD. The study was not intended to define a drought or any similar event through the values of MDD. Instead, it tried to find a correlation among MDDs of all climate stations in Bangladesh through regressions using ML algorithms.

MDDs of the target stations showed a good regression with different MWDs and MDDs of the predictor stations in Bangladesh. ML algorithms were capable to build a fine prediction model of MDD. A Prolonged dry spell or regional drought due to low or no rainfall is objectionable by an agricultural sector^14^. Dryness is the defining feature of a dry spell, thereby allowing the interpretation of a drought. This study can help the agricultural sector to take precautions against periodical dry days in a month. The predicted models were assessed on the basis of *R*^*2*^ and *RMSE*. A very strong regression was found in MDDs of the climate stations. MWDs were also firmly correlated with MDDs which would direct a future study on targeting MWD of the target stations. Response in one year lead time was also satisfactory to predict MDD.

Sensitivity analyses studied the effectiveness of each station to be present in producing desired level of model output. In summary of the sensitivity analysis, it is concluded that a particular station was not highly sensitive for most of the target stations. However, Sylhet and Dinajpur were found sensitive for predicting MDD of Rangpur and Srimangal, respectively. In general, a specific station would not produce much deviation in the model outputs.

Uncertainty analysis assessed the domain of the study data for predicting MDD with a satisfactory level of output. Robustness of the proposed models through Monte Carlo simulation was clearly determined for certain ranges of random input data. Most of the cases, input data could vary with maximum *CV* of 10% to limit the output of the predicted model at a satisfactory level. Figures 6 and 7 depicted the summary of this scenario. However, for Ranpur, Mymensingh, Dinajpur and Bogra, the robustness of the EPGR was sustained up to the random data at *CV* of 0.5.

Several optimized model parameters from the simulation of different ML algorithms in MATLAB are summarized. Tables 4 and 5 present the changes of the optimized parameters of the developed EGPR models for six target stations. The EGPR models with these values of the model parameters can be used for forecasting MDD without lead (Table 4) and with one year lead time (Table 5).

**Table 4:** Optimized parameters of EGPR models for six target stations without any lead.

*Model parameters*	Sylhet	Srimangal	Rangpur	Mymensingh	Dinajpur	Bogra
*LogLikelihood*	− 632.93	− 628.31	− 541.60	− 544.62	− 449.88	− 515.68
*Kernel function: SigmaL*	21.8883	8.2939	68.8375	36.7693	67.6816	32.9028
*Kernel function: SigmaF*	10.4600	6.5132	13.2054	9.7234	11.8457	8.3212
*Beta*	12.1846	19.6351	22.0104	16.9517	20.8954	19.9433
*Sigma*	0.0941	0.0740	0.0655	0.0746	0.0631	0.0634
*ActiveSetMethod*	*Random*	*Random*	*Random*	*Random*	*Random*	*Random*

*SigmaL* length scale for predictors, *SigmaF* signal standard deviation, *Beta* initial value of coefficients, *Sigma* initial value for the noise standard deviation of the Gaussian process model, *ActiveSetMethod* active set selection method, *LogLikelihood* the natural logarithm of the likelihood.

**Table 5:** Optimized parameters of EGPR models in one year lead time.

Model parameters	Sylhet	Srimangal	Rangpur	Mymensingh	Dinajpur	Bogra
*LogLikelihood*	− 677.64	− 660.16	− 610.62	− 627.51	− 597.89	− 605.84
*Kernel function: SigmaL*	8.4501	9.8512	9.9992	11.1110	14.1240	11.3839
*Kernel function: SigmaF*	6.4281	5.9975	5.2892	5.9552	5.7041	5.4368
*Beta*	13.4357	19.0560	19.1886	17.0954	20.4283	19.2318
*Sigma*	1.0688	1.3443	0.6809	0.6821	0.7100	0.6892
*ActiveSetMethod*	*Random*	*Random*	*Random*	*Random*	*Random*	*Random*

The outcome of the study demonstrates the possibility of using MDWD instead of consecutive dry days^32–35^. This approach can be useful for defining dry periods with certain rainfall thresholds. The rainfall threshold used in this study was 2 mm ^27^. This concept can be used for real-time dry day forecasting by reducing computational time, improving water resource management against possible droughts, and reducing the cost of unnecessary field data collection. Hence, the novelty of the study comes from several outcomes using different ML algorithms through the correlation analysis on monthly dry days between different stations and the relationship between monthly dry days and monthly wet days. It demonstrates that ML methods are capable of outperforming current state-of-the-art methods for the prediction of MDD, representing a novel approach of lead-time phenomena with an established path for forecasting MDD.

## Conclusion

MWD and MDWD datasets were prepared based on daily rainfall at all stations in Bangladesh to establish a strong regression with MDD of the six target stations in Northern Bangladesh. The summary of all approaches points out EGPR as the best model among EGPR, BT, MGPR, FT, LSVM and LR. In addition, lead time effort also presented a satisfactory result to forecast MDD for one year ahead.

Uncertainty analyses based on Monte Carlo simulation has established robustness of the developed EGPR model. In summary of the sensitivity analysis, a particular station was not highly sensitive for most of the target stations. Sylhet and Dinajpur were found sensitive for Rangpur and Srimangal, respectively. Hence, sensitivity analysis for this intended procedure and models of the study is less result oriented. The combination of all approaches and the findings with the predictors and responses confirmed the novelty of the study. The outcomes of the study are summarized as:

- EGPR algorithm was able to provide satisfactory model with highest mean *R*^*2*^ of 0.91 and lowest mean *RMSE* of 2.14 among all six algorithms.
- A very good regression was found among MDD and MWD. Hence, dry days with 0–2 mm rainfall have a strong correlation with 10–25 mm and 26–50 mm of rainfall.
- The inclusion of one year lead time also performed very well by EGPR and showed the best response for forecasting MDD.
- EGPR model was assessed its robustness through Monte Carlo simulation. The model is robust up to *CV* of 0.1 for considering random data in a single station and two stations.
- For most of the target stations, no any station is highly sensitive except Sylhet and Dinajpur.

This study provides novel insights into the analysis of monthly dry and wet days in climate research, which may directly or indirectly relate to the actual impacts of droughts. These results could be used in a future study for the definition of a new drought situation with other drought indices based on a strong relationship with monthly dry days. Future studies could seek to establish the relationship between dry events and consecutive dry days compared with different drought indices. More generally, within the broad area of intelligent systems, this study showed that ML algorithms can be applied to establish relationships between dry and wet days.

## Methods

### Study area and data

Bangladesh is prone to natural disasters and extremely vulnerable to climate change^74,75^. Bangladesh extends from 20° 34 N to 26° 38 N and 88° 01 E to 92° 41 E. Except for the hilly southeast, the majority of the country is characterized by low-lying plains situated on deltas of large rivers flowing from the Himalayas. The country is surrounded by the Meghalaya Plateau in the north, the lofty Himalayas lying farther to the north, the Assam Hills in the east, and the Bay of Bengal in the south. Located in a tropical monsoon region, the climate of Bangladesh is characterized by moderately warm temperatures and high humidity with marked seasonal variations in rainfall.

The four recognized seasons are a hot, humid summer from March to May, a wet, warm, and rainy monsoon season from June to September, autumn from October to November, and a dry winter from December to February^76–78^. January is the coldest month, with an average temperature of 18.1 °C, while May is the hottest month with an average temperature of 28.7 °C.

In the summer, the mean temperature gradient leans towards the northeast (cooler) from the southwest (warmer); in contrast, the winter mean temperature gradient is oriented towards the north (cooler) from the south (warmer). Rainfall in Bangladesh mostly occurs in the monsoon, induced by weak tropical depressions that are brought from the Bay of Bengal into Bangladesh by wet monsoon winds^77^. More than 75% of the rainfall in Bangladesh occurs during the monsoon season. The daily rainfall in different stations shows a huge rainfall variation in between stations and seasons. Due to reduced or no rainfall, regional droughts currently affect approximately 2.5 million and 1.2 million ha of agricultural land in a year in the wet and dry seasons, respectively^14^. Hence, there would exist a better correlation in terms of varied rainfall magnitudes between stations on a monthly scale or a seasonal scale to deal with dry periods or droughts and there might have better directions to be used in the agriculture sector.

**Figure 8:**
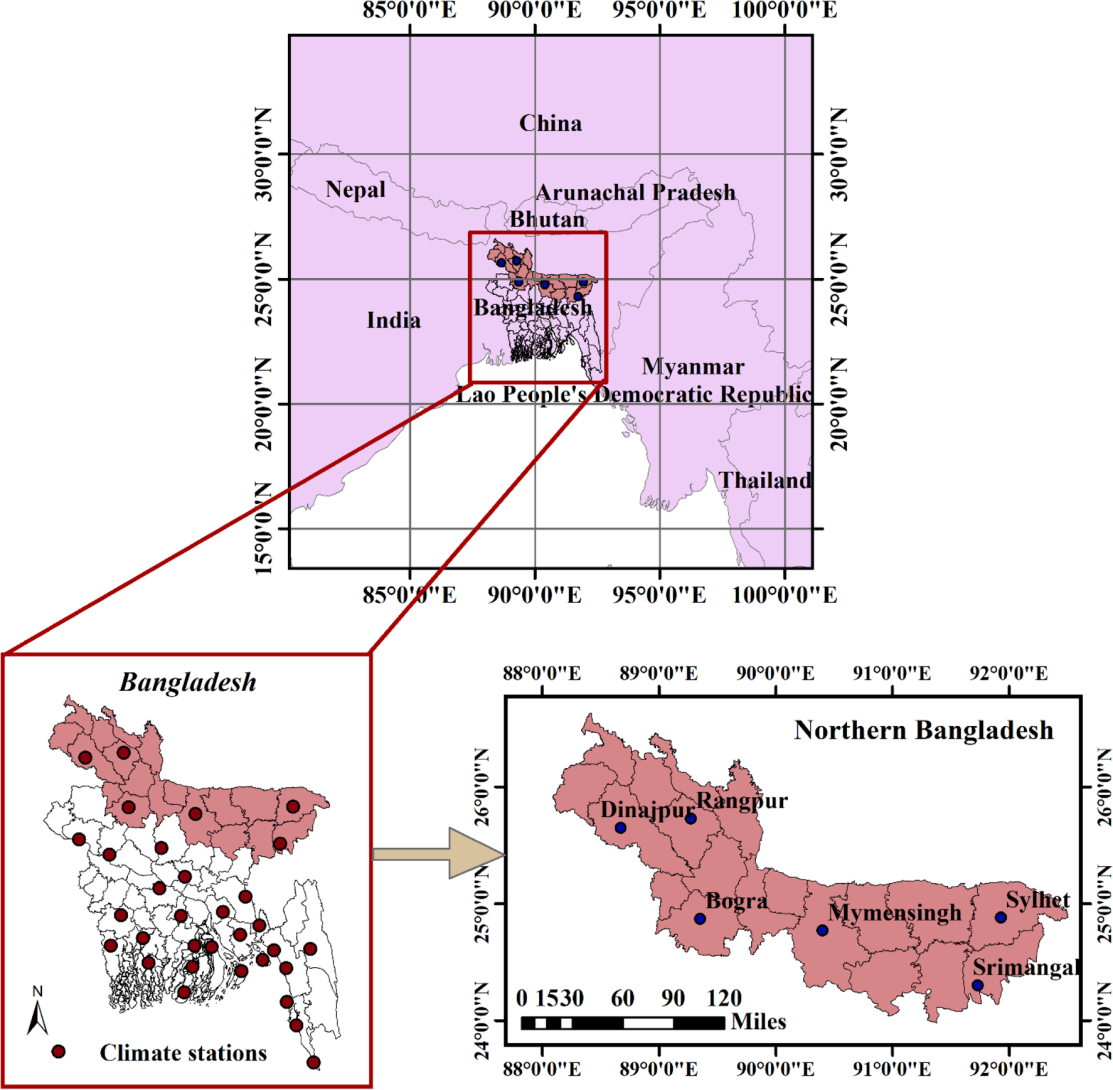
Geolocation of study area with six target stations.

Figure 8 shows 27 rain gauge stations with rainfall records for more than 30 years (1982–2016) operated by the Bangladesh Meteorological Department (BMD). To predict monthly dry days (MDD), we selected only six target stations (Sylhet, Srimangal, Rangpur, Dinajpur, Bogra, and Mymensingh) located in Northern Bangladesh.

A rainfall threshold of 2 mm on a daily scale was used to characterize a dry day and a sample in Table 6 shows monthly cumulative dry days. MDD was defined as the frequency of dry days in a month as elaborated in Table 6. Details of the custom datasets prepared from daily rainfall are listed in Table 7.

**Table 6:** Calculation of dry days when a day has a rainfall less than 2 mm.

Year	Month	Day	Mymensingh, mm	Dry days	Dhaka.mm	Dry days	Faridpur, mm	Dry days
1982	4	1	0	1	0	1	0	1
1982	4	2	0	1	0	1	0	1
1982	4	3	0	1	0	1	0	1
1982	4	4	0	1	0	1	0	1
1982	4	5	0	1	0	1	0	1
1982	4	6	0	1	7	0	13	0
1982	4	7	0	1	8	0	3	0
1982	4	23	2	1	5	0	0	1
1982	4	24	0	1	0	1	0	1
1982	4	25	6	0	11	0	0	1
1982	4	26	2	1	7	0	24	0
1982	4	27	0	1	12	0	5	0
1982	4	28	33	0	11	0	22	0
1982	4	29	0	1	0	1	0	1
1982	4	30	21	0	0	1	3	0
MDD in April 1982			**Mymensingh**	23	**Dhaka**	17	**Faridpur**	18

**Table 7:** Three datasets in this study to predict MDD at six target stations.

Monthly dry days (MDD)	Monthly wet days (MWD)	Monthly dry and wet days (MDWD)
MDD is the summation of dry days in a month where a day is classified as a dry day if daily rainfall is between 0 and 2 mm	MWD is the combination of light wet days (LWD) and average wet days (AWD). A day is classified as LWD if daily rainfall is from 10 to 25 mm, and as AWD if daily rainfall lies between 26 and 50 mm. The MWD dataset comprised data from 54 stations (27 stations for each LWD and AWD). These data are used as predictors and the MDD at each target station is the response	MDWD is the combination of MDD and MWD. The dataset comprised data from 81 stations (27 stations for each MDD, LWD, and AWD)

### Study procedure

After preparing the datasets, the study used Regression Learner toolbox in MATLAB and performed the simulation of the proposed ML models. The study has two perspectives. In first perspective, the predictor stations were used to predict the MDD of the target stations without any lead time whereas in second perspective, the predictor stations were utilized to predict MDD of one year ahead.

**Figure 9:**
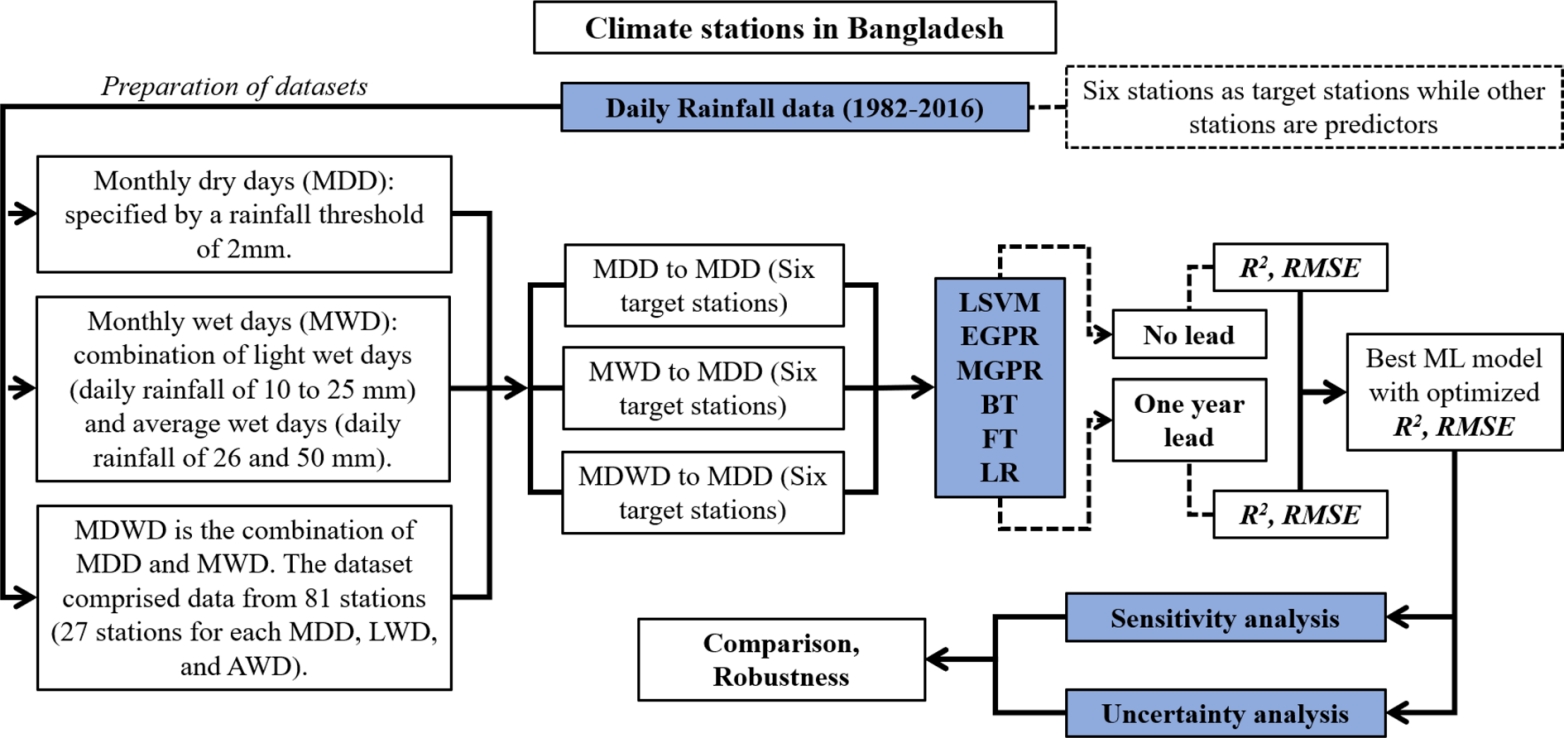
Procedural flow chart of the materials and methods in this study.

The best model was chosen on the basis of optimized values of *R*^*2*^ and *RMSE*. Then sensitivity and uncertainty analysis were performed to establish the robustness of the developed model. The detail procedure of the study is presented through Fig. 9.

### Prediction of MDD

The main objective of this study was to build regression models for the prediction of MDD at six target stations using different rainfall stations as predictors and several ML algorithms.

#### ML algorithms

ML algorithms employ various statistical, probabilistic, and optimization methods to learn from past experiences and detect useful patterns from large, unstructured, and complex datasets. The ML algorithms used in this study were linear regression (LR) models, regression trees (RT), support vector machines (SVM), Gaussian process regression (GPR) models, and bagged trees (BT).

#### LR

Linear regression^79^ is a statistical modeling technique used to describe a continuous response variable as a function of one or more predictor variables. It can help understand and predict the behavior of complex systems or analyze experimental, financial, and biological data. Linear regression was used to create a linear model that describes the relationship between a dependent variable y (also called the response) as a function of one or more independent variables Xi (called the predictors). The general equation for a linear regression model is:

1 $$y = \beta_{0} + \sum \beta_{i} X_{i} + \epsilon_{i}$$

where *β* represents the linear parameter to be computed and *ϵ* represents the error term.

All four linear regression models (linear, interactive linear, robust linear, and stepwise linear) have easy interpretability, but linear and robust linear models have very low flexibility. The regression learner uses the *fitlm* function to train linear, interactive linear, and robust linear models. The app uses the *Stepwiselm* function to train stepwise linear models.

#### RT

A regression tree is built through a process known as binary recursive partitioning, which is an iterative process that splits data into partitions or branches^80^. Then, each partition is split into smaller groups as the method moves up each branch. Initially, all records in the training set (pre-classified records that are used to determine the structure of the tree) are grouped into the same partition. The algorithm then begins to allocate data into the first two partitions or branches using every possible binary split in every field. The algorithm then selects the split that minimizes the sum of the squared deviations from the mean in the two separate partitions. This splitting rule is then applied to each new branch. This process continues until each node reaches a user-specified minimum node size and becomes a terminal node. If the sum of the squared deviations from the mean in a node is zero, then that node is considered to be a terminal node even if it has not reached the minimum size.

#### SVM

SVM, a supervised learning model, was introduced through different studies^81–83^. The basic idea of SVM is to find a hyperplane in a high-dimensional space to separate data using the structural risk minimization (SRM) principle based on the Vapnik–Chervonenkis (VC) dimension. For a classification task, SVM is a binary classification model. The binary classifier assumes that there are two classes in the task, and that each class is well identified by the decision surface. A sequence of binary classifiers can be used to handle multiclass tasks. For example, this study used two classes of flags. An event was classified as 1, and a non-event (background) was classified as 0.

The general idea of the SVM can be summarized as follows: suppose a set of datasets with k samples, {*x*_*i*_, *y*_*i*_}, *i* = 1, …, *k*, where *x* ∈ R_n_ is an *n*-dimensional vector and *y* ∈ {− 1, + 1} denotes the corresponding class label. The SVM calculates a hyperplane with the maximum margin by solving the following equation:

2 $$\begin{aligned} & min\frac{1}{2}\left( w \right)^{T} \cdot w + C\mathop \sum \limits_{i = 1}^{k} \xi_{i} \\ & {\text{s}}.{\text{t}}.\;\; y_{i} (w^{T} \cdot \varphi \left( {w_{i} } \right) + b) \ge 1 - \xi_{i} ,\quad \xi_{i} \ge 0, i = 1, \ldots ,k \\ \end{aligned}$$

where ∅(*x*_*i*_) maps the input space to the feature space. *C* > 0 is a penalty factor that controls the trade-off between the minimization of the classification error and maximization of the margin. $w$, $b$, and $\xi$ were optimized during the training phase.

The optimal decision surface can be determined by introducing Lagrange multipliers, and the classification function is represented as

3 $$f\left( x \right) = sgn \left( {y_{i} a_{i} k\left( {x_{i} , x} \right) + b^{*} } \right)$$

where *a*_*i*_ is the support vector, *b*^∗^ is the bias, and *k* (*x*_*i*_, *x*) = 〈∅(*x*_*i*_), ∅(*x*)〉 is the kernel function.

#### GPR

GPR models are nonparametric kernel-based probabilistic models^84^. A GPR model can be trained using the *fitrgp* function. Consider the training set {(*x*_*i*_, *y*_*i*_); *i* = 1, 2, …, *n*}, where *x*_*i*_ ∈ ℝ^*d*^ and *y*_*i*_ ∈ ℝ, drawn from an unknown distribution. A GPR model addresses the question of predicting the value of a response variable *y*_*new*_, given a new input vector *x*_*new*_ and training data. A linear regression model has the form

4 $$y = x^{T} \beta + \varepsilon$$

where *ε* ∼ *N* (0, *σ*^2^). The error variance *σ*^2^ and coefficients *β* are estimated from the data. A GPR model explains the response by introducing latent variables, *f* (*x*_*i*_), *i* = 1, 2, …, *n*, from a Gaussian process (GP) and explicit basis functions, *h*. The covariance function of the latent variables captures the smoothness of the response, and the basic functions project the inputs *x* onto a *p*-dimensional feature space.

A GP is a set of random variables, such that any finite number of them have a joint Gaussian distribution. If {*f*(*x*), *x* ∈ ℝ^*d*^} is a GP, then given *n* observations of *x*_1_, *x*_2_, *…*, *x*_n_, the joint distribution of the random variables *f(x*_1_*)*, *f(x*_2_*)*, *…*, *f(x*_*n*_*)* is Gaussian. A GP is defined by its mean function *m*(*x*) and covariance function *k* (*x*, *x*). In other words, if {*f*(*x*), *x* ∈ ℝ^*d*^} is a Gaussian process, then *E* (*f*(*x*)) = *m*(*x*) and *Cov*[*f*(*x*), *f*(*x*)] = *E*[{*f*(*x*) − *m*(*x*)}{*f*(*x*) − *m*(*x*)}] = *k* (*x*, *x′*). There are four types of GPR models: rational quadratic, squared exponential, Matern 5/2, and exponential. Each type of model has a hard interpretability and automatic flexibility to fit datasets.

Exponential GPR*.* One can specify the exponential kernel function using the “KernelFunction,” “exponential” name–value pair argument. This covariance function is defined as follows:

5 $$k (x_{i} ,x_{j} |\theta ) = \sigma_{f}^{2} exp\left( { - \frac{r}{{\sigma_{l} }}} \right)$$

where *σl* is the characteristic length scale and

6 $$r = \surd \left( {\left( {x_{i} - x_{j} } \right)^{T} \left( {x_{i} - x_{j} } \right)} \right)$$

is the Euclidean distance between *x*_*i*_ and *x*_*j*_.

#### Matern 5/2 GPR

One can specify the Matern 5/2 kernel function using the “Kernel Function,” “matern52” name–value pair argument. The Matern 5/2 covariance function is defined as

7 $$k \left( {x_{i} ,x_{j} } \right) = \sigma_{f}^{2} \left( {1 + \frac{\sqrt 5 r}{{\sigma_{l} }} + \frac{{5r^{2} }}{{3\sigma_{l}^{2} }}} \right)\exp \left( - \frac{\sqrt 5 r}{{\sigma_{l} }}\right)$$

where

8 $$r = \surd \left( {\left( {x_{i} - x_{j} } \right)^{T} \left( {x_{i} - x_{j} } \right)} \right)$$

is the Euclidean distance between *x*_*i*_ and *x*_*j.*_

#### BT

Ensemble learning is currently a primary and popular research direction in data mining and ML. By training many base learning systems, aggregating these base learning systems, and using multiple versions of the learning system to solve the same problem, the generalization ability of a learning system can be improved significantly. Ensemble learning is regarded as a computing technique that has broad prospects for many applications. Currently, there are many ensemble learning algorithms, including bagging, boosting, and subspace, whereof bagging is a well-known algorithm.

The bagging algorithm^85^ was first proposed by Breiman in 1996. A training set *D* consists of data {(*y*_*i*_, *x*_*i*_), *i* = 1, …, *N*}, where *x*_*i*_ is an instance and *y*_*i*_ is a label of class label set *Y* whose amount is *k*. A classifier *φ*(*x*, *D*) is built using a given method. If an instance *x* is input with an unknown class label, a class label *y* can be predicted with *φ*(*x*, *D*). Suppose that there is a training set sequence {*D*_1_, …, *D*_*m*_}, where the number of instances of *D*_*i*_ is the same as that for *D*. *N* instances in *D*_*i*_ are randomly selected from *D* by bootstrap sampling with replacement. The value of *m* is set in advance; for instance, it can be set to 50. The learning mission uses {*D*_1_, …, *D*_*m*_} to obtain a better classifier than classifier *φ*(*x*, *D*), which is learned from a single training set *D*. If *y* is numerical, an obvious procedure is to replace *φ*(*x*, *D*) with *E* (φ (*φ*(*x*, *D*)), that is, by the average of *φ*(*x*, *D*_*k*_) over *k* (1 ≤ *k* ≤ *m*), where *E*(*φ*(*x*, *D*)) denotes the expectation over *D*. If *y* is nominal, then one method is to aggregate the results of *φ*(*x*, *D*_*k*_) by voting. This aggregation method is called “bootstrap aggregating” or “bagging.” The bagging ensemble technique has been successfully used in civil engineering applications for the prediction of material properties ^86^.

### One-year lead time

Given early warnings with sufficient lead time, water resource management authorities and other civil protection bodies can exercise caution and take preventive measures to mitigate the impacts of any climatic event, such as droughts, floods, or cyclones. Several studies have been undertaken by the Multi-hazard Mitigation Council^87^ and United Nations Development Program^88^, with the money spent on emergency response being far more effective and less costly than money spent on recovery efforts. A well-built early dry period or drought warning system could inform decision makers in agriculture and water resource management bodies, enabling the establishment of preventive measures.

This study incorporates a new approach to verify the performance of ML techniques in forecasting MDD by considering a shift in the target time series. The shift is assumed to be a one-year lead. Six ML techniques, namely BT, EGPR, MGPR, LSVM, FT, and LR, were used to perform the simulation. Three previous approaches and their corresponding predictors were used in this scenario to find effective predictors to produce the best forecasting model. To do this, the training periods for the predictors and response were from 1982 to 2003 and 1983 to 2004, respectively. The testing period for the predictors was from 2004 to 2016; hence, values were forecasted from 2005 to 2017.

### Uncertainty analysis

Monte Carlo (MC) simulation is used to perform the uncertainty analysis of the proposed model in order to demonstrate the methodology robustness with respect to uncertainties in different input data. Random data for every input station is generated and the new data set is used to check the performance of the model comparing with actual data. Different ranges of coefficients of variation are used to generate random data. Monte Carlo simulation was used to perform uncertainty in different water model parameters^68,69^ and checked the robustness of the proposed models. Different coefficients of variation were used ^68,69^ to range the random data having specific mean of each input data. In this study, same procedure of uncertainty analysis is followed by generating random data, using different coefficient of variations and specific mean. The uncertainty analysis worked out in consideration of two cases. At first, data of a single station would be random with uncertainty at different coefficients of variation (*CV*). Secondly, any two stations would be random within different *CVs*. Here, 0.01, 0.05, 0.1, 0.5, 1 and 2 are *CVs* incorporated to do the simulation.

### Sensitivity analysis

**Figure 10:**
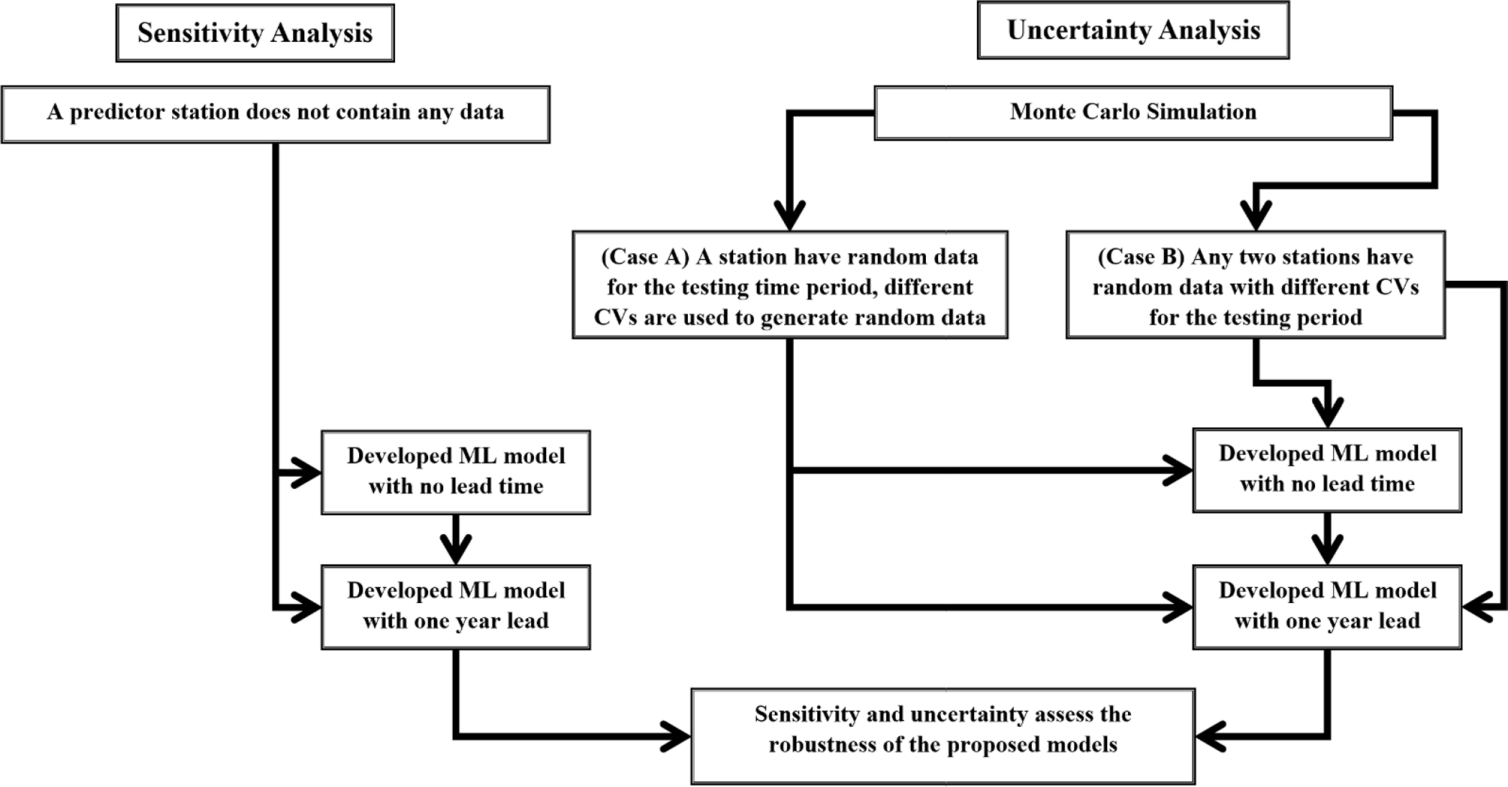
Flow chart of sensitivity and uncertainty analysis in this study.

A sensitivity analysis is a technique used to determine how significant any input parameter is to reach to a desired level of output. At first, it was assumed that a single station would not contain any data for the whole testing period of analysis. To perform sensitivity analysis, a scenario was assumed that a station does not have any study data in the testing period. Significance of the station parameters in model validation is usually checked through this process. Then of course the sensitivity analysis finds which station is more or less sensitive to the developed model of MDD. Figure 10 demonstrates the procedure of the sensitivity and uncertainty analysis.

### Performance indicators

*R*^*2*^ and *RMSE* were the two performance indicators used to define the efficiency of the training and testing models. Both *RMSE* and *R*^*2*^ quantify how well a regression model fits the dataset. The *RMSE* indicates how well a regression model can predict the value of the response variable in absolute terms, while *R*^*2*^ indicates how well a model can predict the value of the response variable in percentage terms.

9 $$R^{2} = \frac{{\mathop \sum \nolimits_{i = }^{n} (x_{i} - \overline{x})^{2} (y_{i} - \overline{y})^{2} }}{{\mathop \sum \nolimits_{i = }^{n} (x_{i} - \overline{x})^{2} \mathop \sum \nolimits_{i = }^{n} (y_{i} - \overline{y})^{2} }}$$

10 $$RMSE = \frac{{\mathop \sum \nolimits_{i = }^{n} (x_{i} - y_{i} )^{2} }}{n}$$

where *x*_*i*_ and *y*_*i*_ are the actual and predicted values, respectively, $\overline{x}$ and $\overline{y}$ are the average actual and predicted values, respectively, and *n* is the number of values.

## Supplementary Information

Supplementary Information.

## Data Availability

The datasets generated and/or analyzed during the current study are not publicly available due to inclusion of the datasets to the subsequent studies and involves other unpublished ancillary works that are currently under analysis, but are available from the corresponding author on reasonable request.
