# Supplementary material for: Prediction of monthly dry days with machine learning algorithms: a case study in Northern Bangladesh
Source: Sci Rep. 2022 Nov 16;12:19717. doi: 10.1038/s41598-022-23436-x (PMC9668981; doi:10.1038/s41598-022-23436-x)
Supplement: Supplementary file 1 — Supplementary Information. [file 41598_2022_23436_MOESM1_ESM.pdf]

Supplementary Materials

Prediction of Monthly Dry Days with Machine Learning Algorithms: A Case Study in Northern Bangladesh

Contents:

Figure S1: Time series dataset of monthly dry days (MDD) for all stations

Figure S2.a. Actual and predicted MDD using all ML models when targets are: A=Sylhet, B=Srimangal, C=Rangpur

Figure S2.b. Actual and predicted MDD using all ML models when targets are D=Mymensingh, E=Dinajpur and F=Bogra.

Figure S3. Forecasted MDD using EGPR for the stations Sylhet, Srimangal, Rangpur, Mymensingh, Dinajpur, and Bogra

Figure S4. Sensitivity of different stations for predicting MDD of six target stations: A=Sylhet, B=Srimangal, C=Rangpur, D=Mymensingh, E=Dinajpur and F=Bogra.

Figure S5. Variation of R^2 values when a station has random data with different CV: A=Sylhet, B=Srimangal, C=Rangpur, D=Mymensingh, E=Dinajpur and F=Bogra.

Figure S6. Variation of *RMSE* values when a station has random data with different CV: A=Sylhet, B=Srimangal, C=Rangpur, D=Mymensingh, E=Dinajpur and F=Bogra.

Figure S7. Variation of R^2 values when two stations have random data with different CV: A=Sylhet, B=Srimangal, C=Rangpur, D=Mymensingh, E=Dinajpur and F=Bogra.

Figure S8. Variation of *RMSE* values when two stations have random data with different CV: A=Sylhet, B=Srimangal, C=Rangpur, D=Mymensingh, E=Dinajpur and F=Bogra.

Table S1: Variation in R^2 for training and testing datasets using *(a) MDD* to *MDD*, *(b) MWD* to *MDD*, and *(c) MDWD* to *MDD*

Table S2: Variation in *RMSE* for training and testing datasets using *(a) MDD* to *MDD*, *(b) MWD* to *MDD*, and *(c) MDWD* to *MDD*

Table S3. R^2 *& RMSE* of the ML models for the approaches *i) MDD to MDD ii) MWD to MDD & iii) MDWD to MDD* using testing dataset

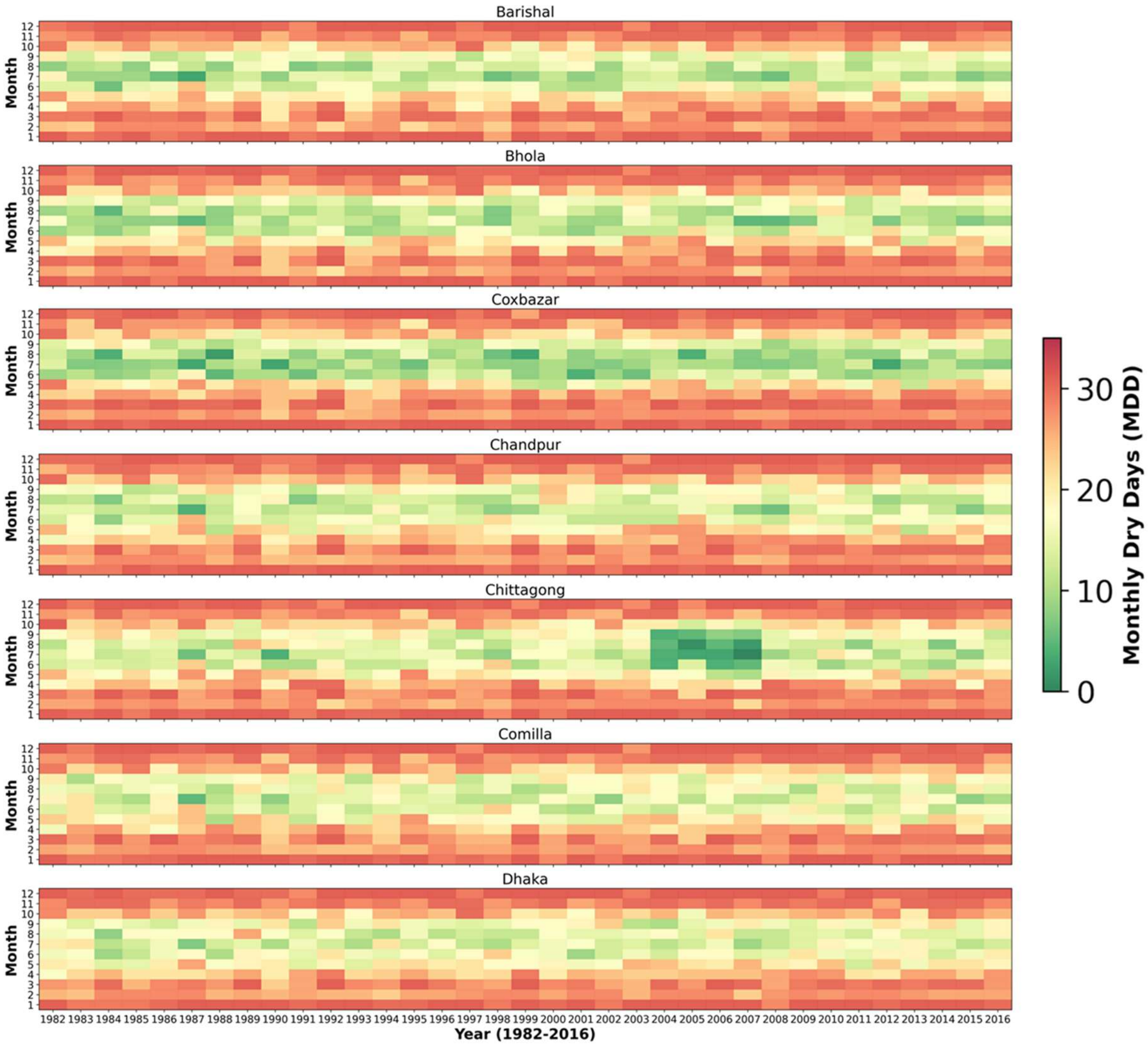

Figure S1. Time series dataset of monthly dry days (MDD) for all stations

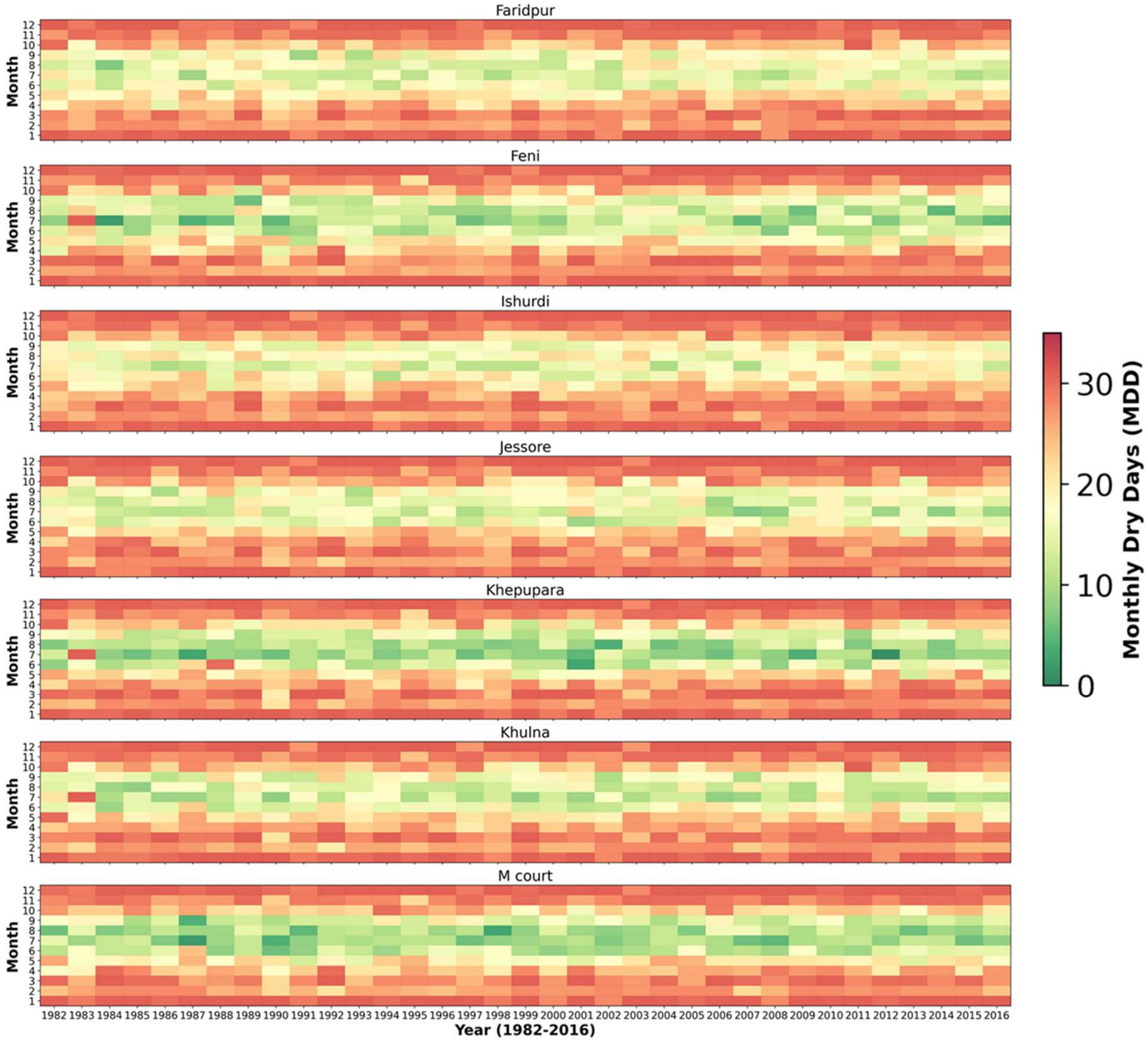

Figure S1. (continued)

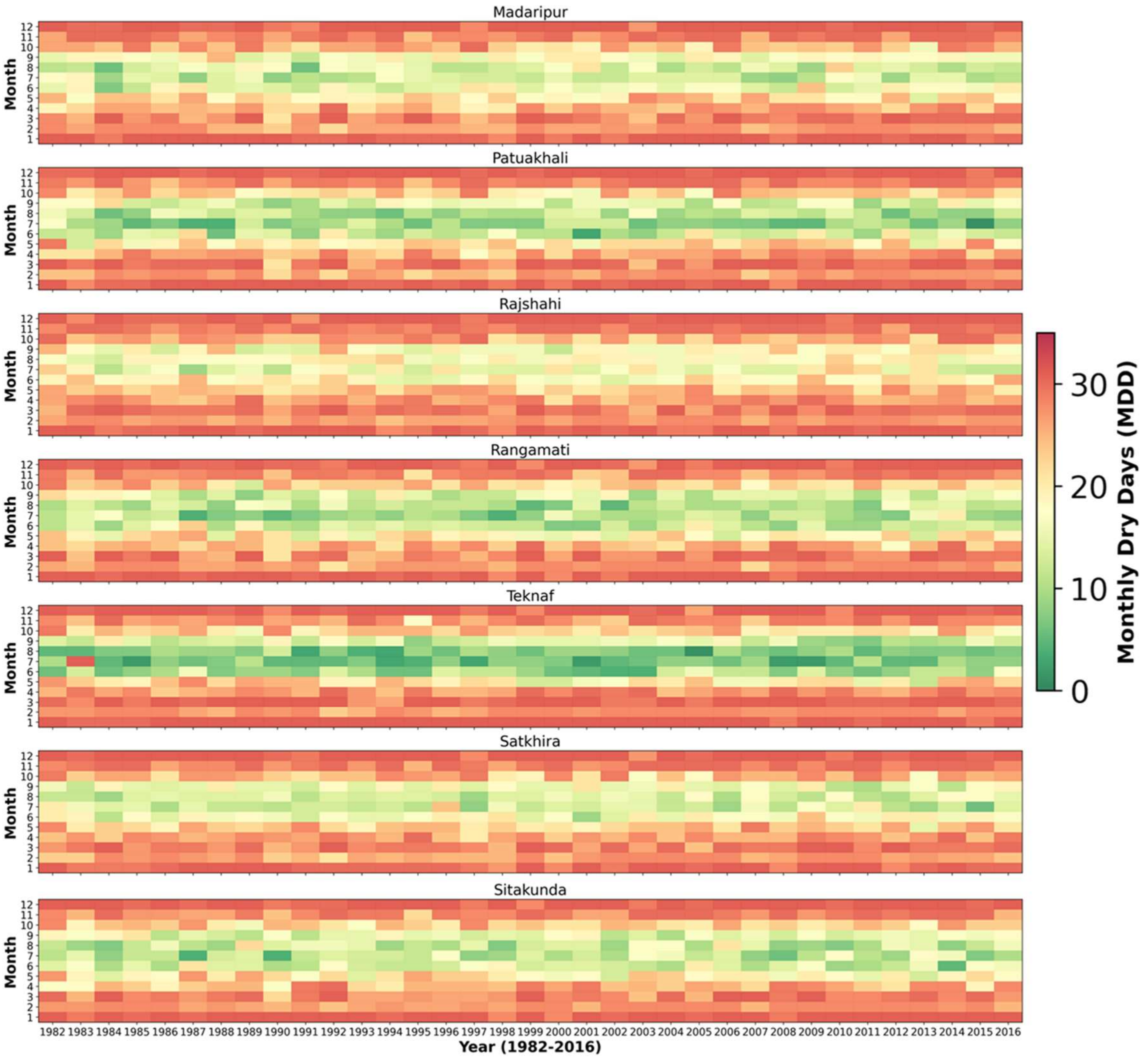

Figure S1. (continued)

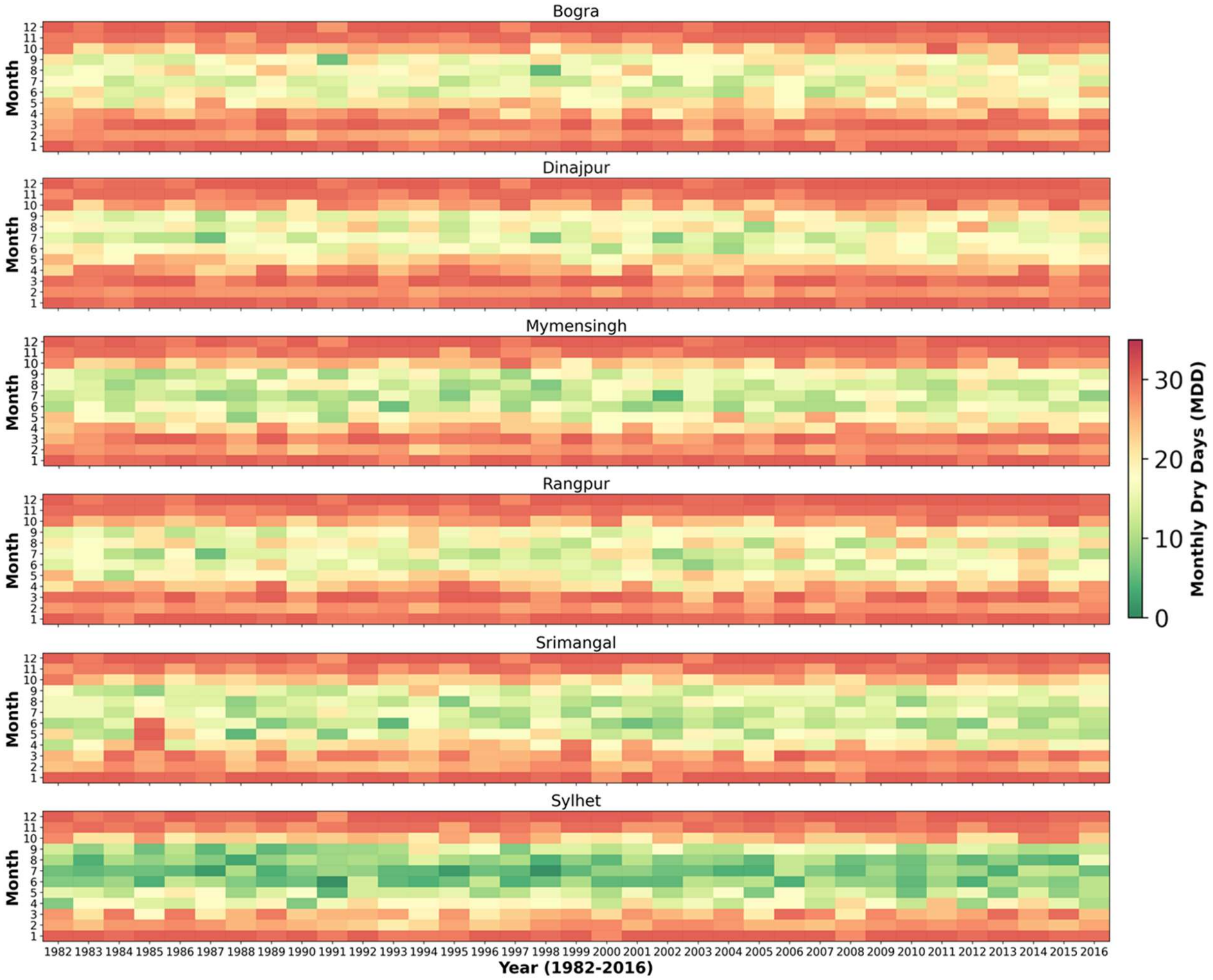

Figure S1. (continued)

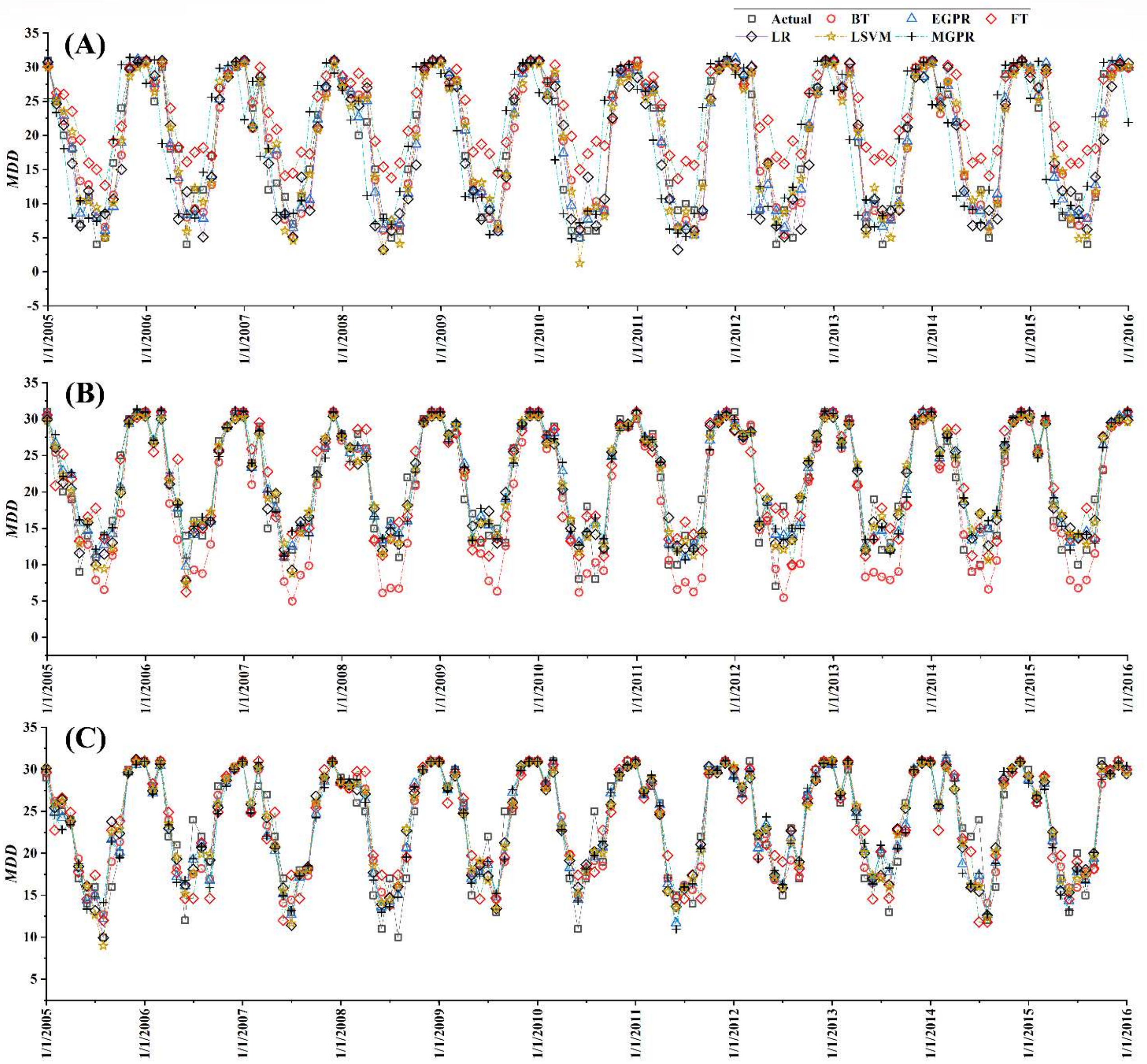

Figure S2.a. Actual and predicted MDD using all ML models when targets are: A=Sylhet, B=Srimangal, C=Rangpur

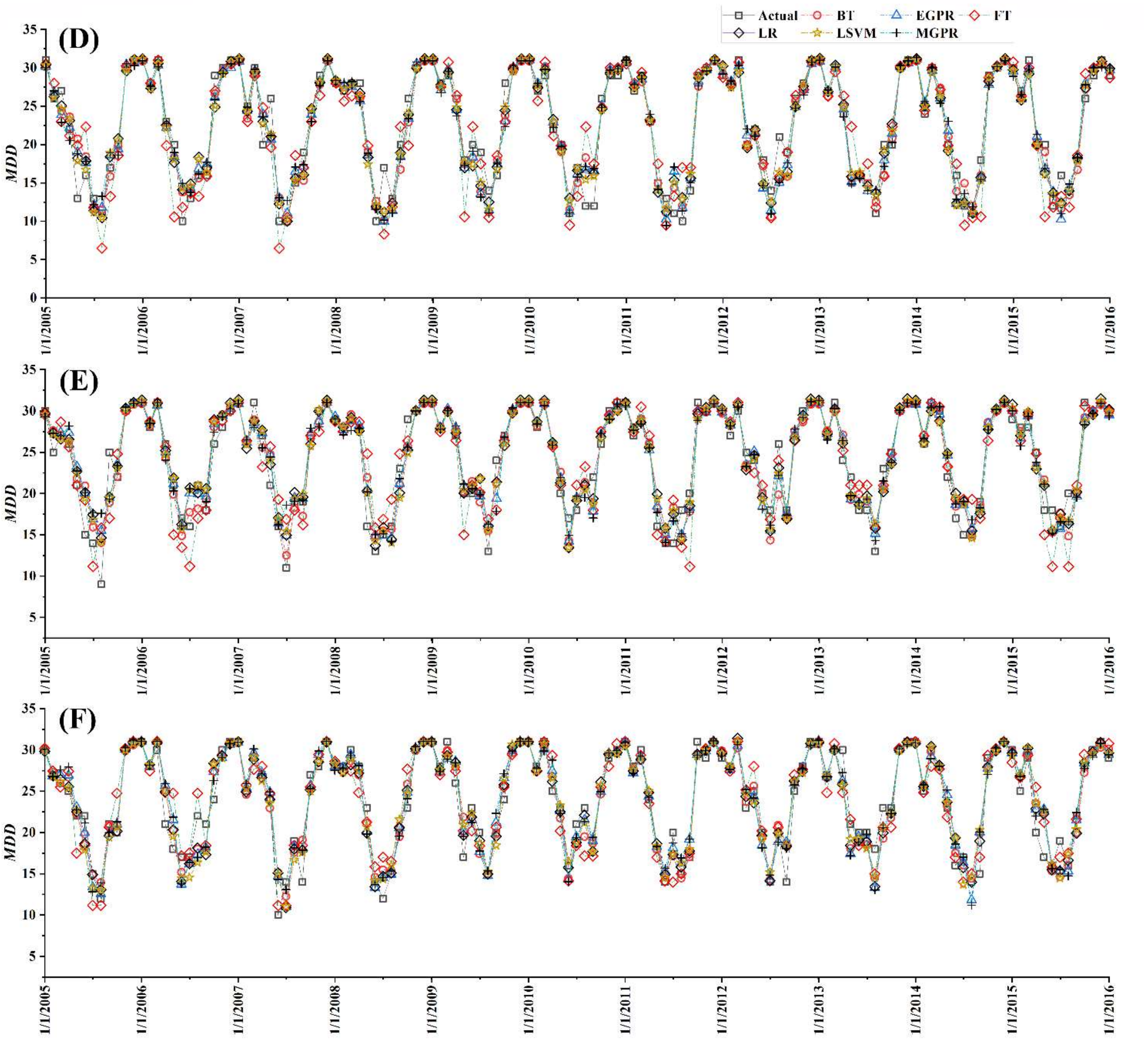

Figure S2.b. Actual and predicted MDD using all ML models when targets are: D=Mymensingh, E=Dinajpur and F=Bogra.

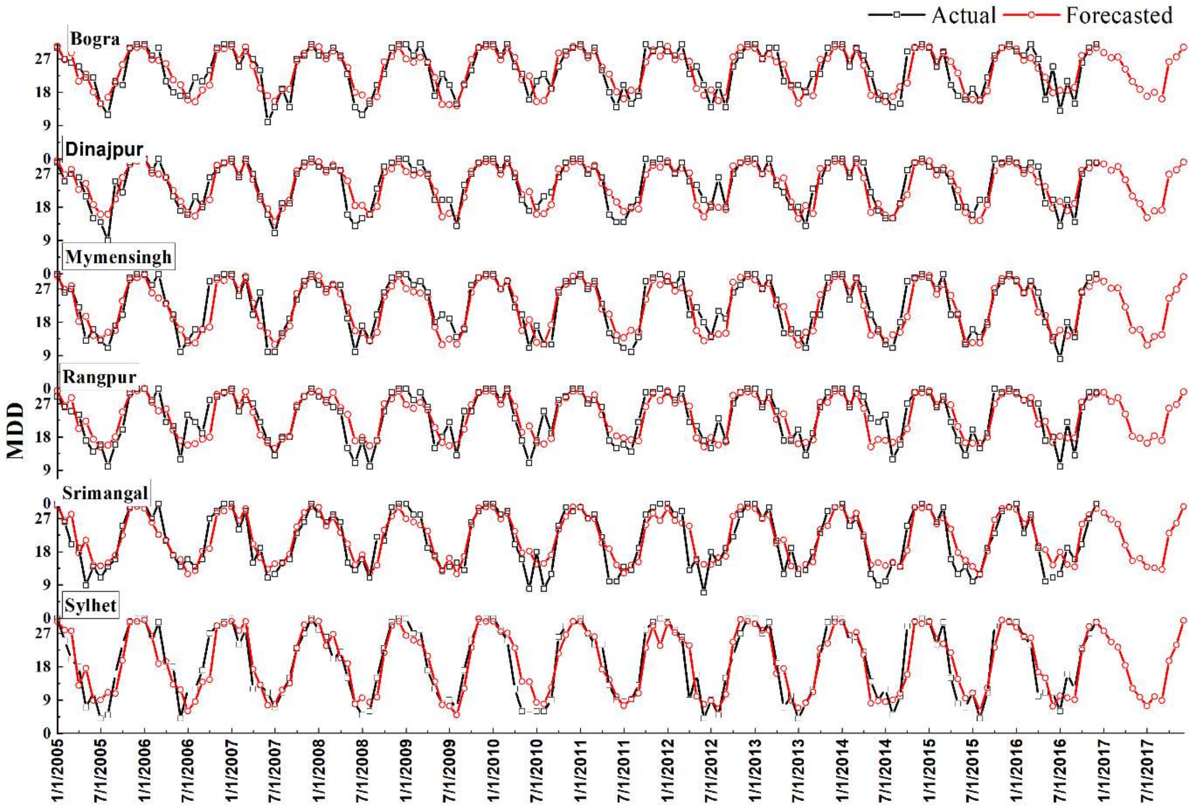

Figure S3. Forecasted MDD using EGPR for the stations Sylhet, Srimangal, Rangpur, Mymensingh, Dinajpur, and Bogra

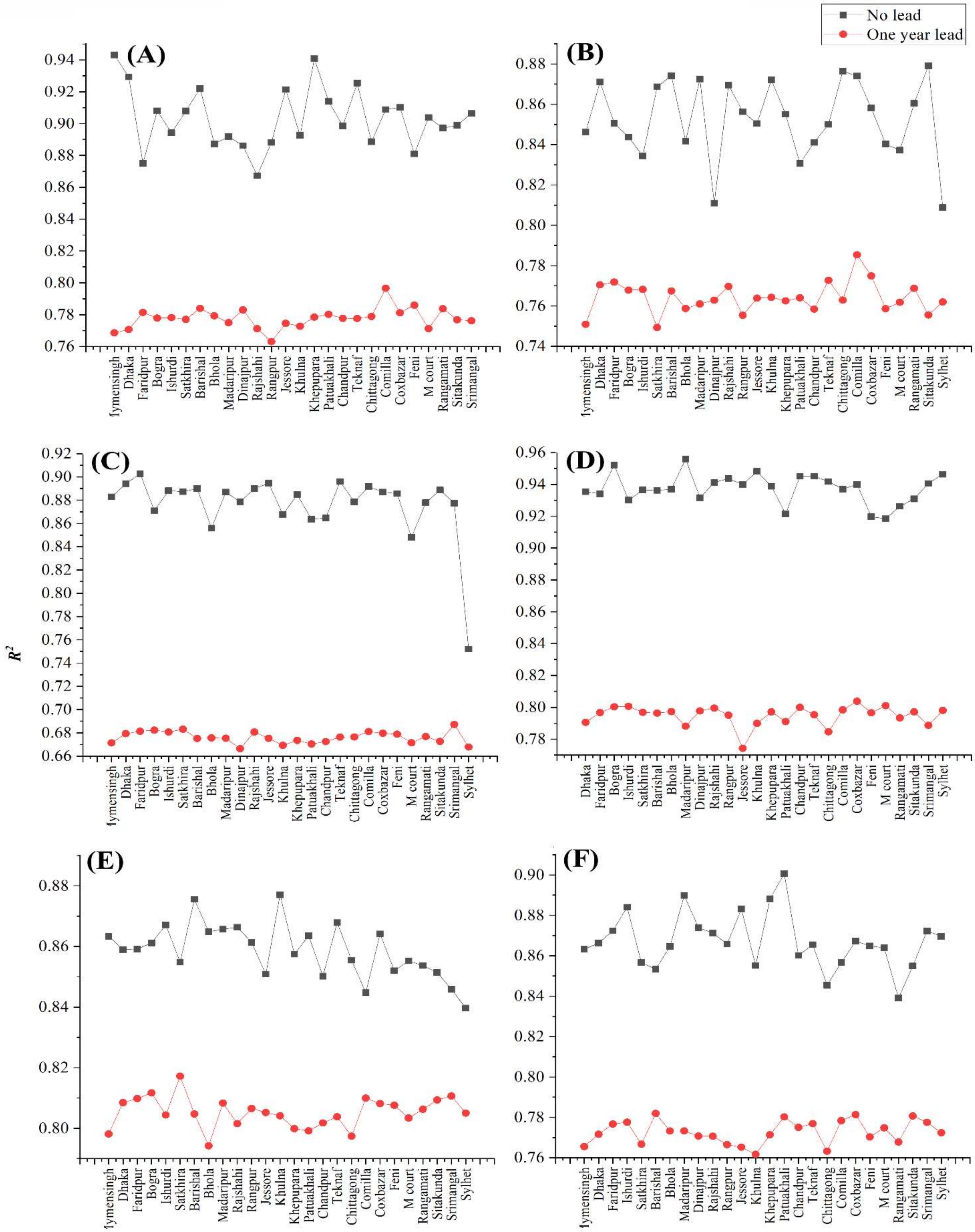

Figure S4. Sensitivity of different stations for predicting MDD of six target stations: A=Sylhet, B=Srimangal, C=Rangpur, D=Mymensingh, E=Dinajpur and F=Bogra.

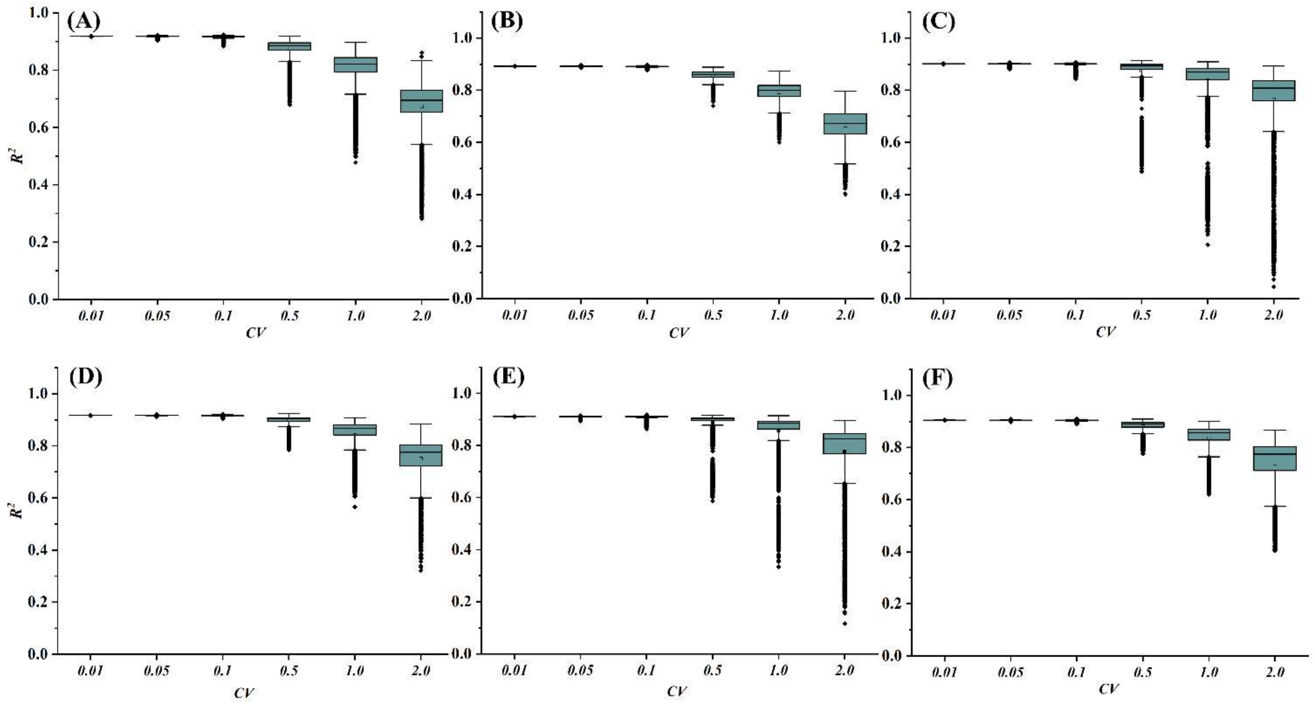

Figure S5. Variation of R^2 values when a station has randomness with different CV: A=Sylhet, B=Srimangal, C=Rangpur, D=Mymensingh, E=Dinajpur and F=Bogra.

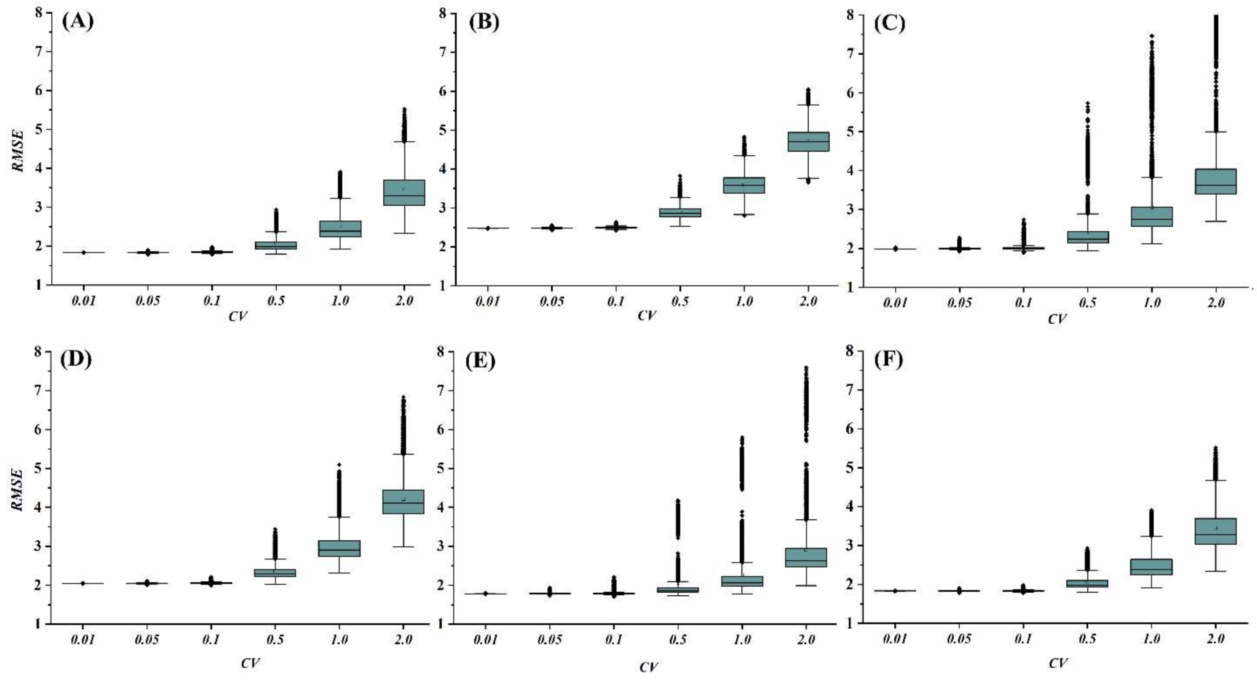

Figure S6. Variation of *RMSE* values when a station has randomness with different CV: A=Sylhet, B=Srimangal, C=Rangpur, D=Mymensingh, E=Dinajpur and F=Bogra.

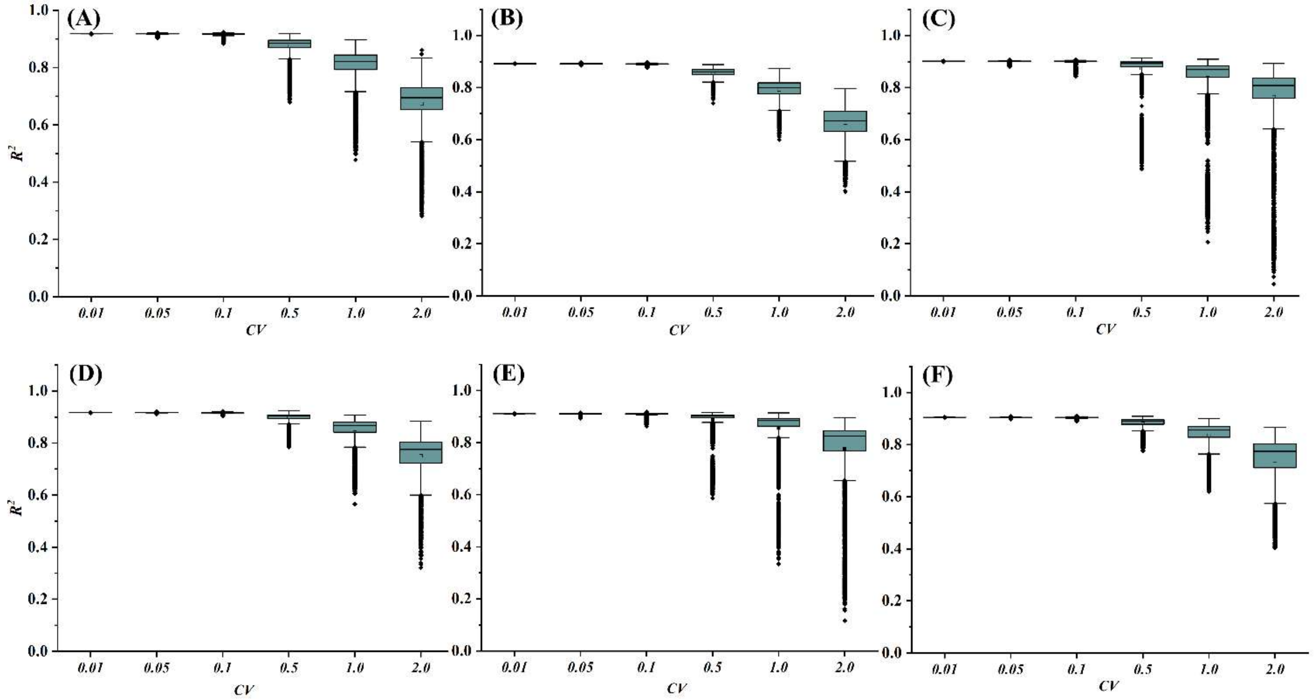

Figure S7. Variation of R^2 values when two stations had random data with different CV: A=Sylhet, B=Srimangal, C=Rangpur, D=Mymensingh, E=Dinajpur and F=Bogra.

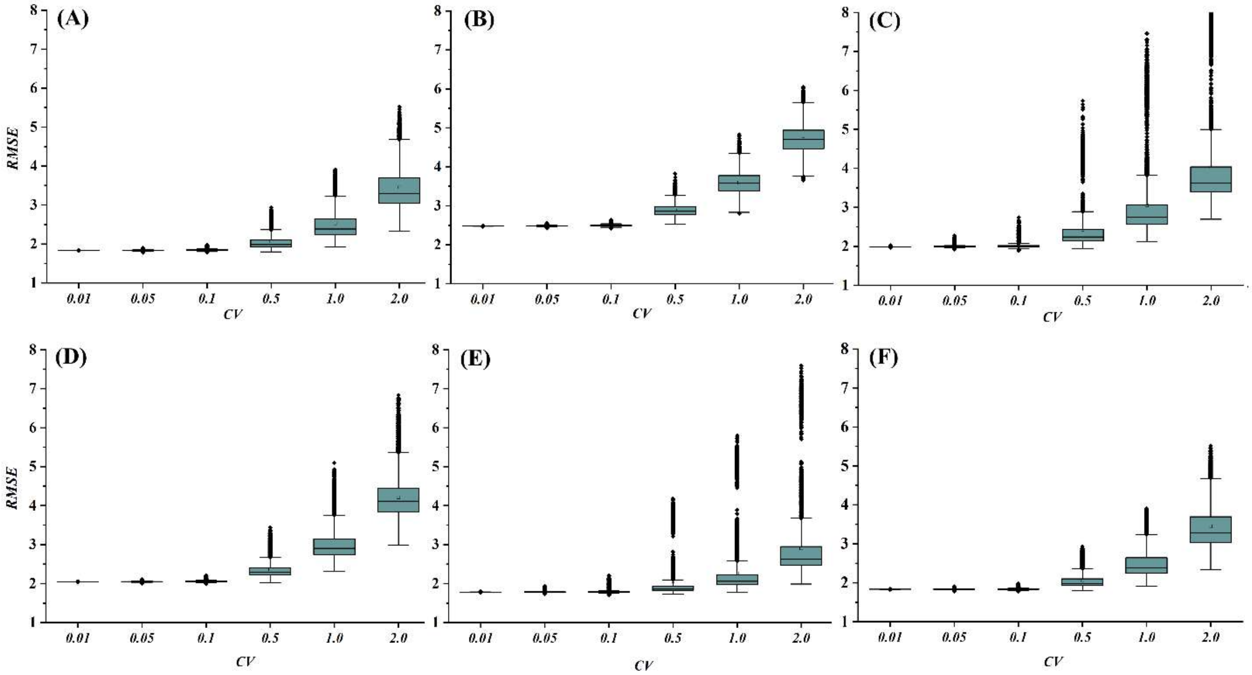

Figure S8. Variation of *RMSE* values when two stations have randomness with different CV: A=Sylhet, B=Srimangal, C=Rangpur, D=Mymensingh, E=Dinajpur and F=Bogra.

Table S1. R^2 of the ML models for the approaches *i) MDD to MDD ii) MWD to MDD & iii) MDWD to MDD*

Approach	ML Model	Training: Sylhet	Training: Srimangal	Training: Rangpur	Training: Mymensingh	Training: Dinajpur	Training: Bogra	Training: *Mean*	Testing: Sylhet	Testing: Srimangal	Testing: Rangpur	Testing: Mymensingh	Testing: Dinajpur	Testing: Bogra	Testing: *Mean*
**MDD to MDD**	***BT***	0.96	0.95	0.95	0.95	0.95	0.92	***0.95***	0.92	0.90	0.9	0.88	0.89	0.87	***0.89***
	EGPR	1.00	1.00	1.00	1.00	1.00	1.00	***1.00***	0.92	0.91	0.91	0.90	0.92	0.89	***0.91***
	MGPR	0.99	0.99	1.00	0.99	0.99	0.99	***0.99***	0.91	0.90	0.88	0.88	0.92	0.88	***0.90***
	LSVM	0.94	0.92	0.93	0.91	0.91	0.86	***0.91***	0.94	0.90	0.91	0.89	0.91	0.89	***0.91***
	FT	0.97	0.96	0.97	0.96	0.96	0.94	***0.96***	0.83	0.87	0.82	0.8	0.87	0.83	***0.84***
	LR	0.94	0.92	0.94	0.91	0.92	0.86	***0.92***	0.93	0.91	0.91	0.89	0.90	0.9	***0.91***
**MWD to MDD**	***BT***	0.95	0.92	0.92	0.93	0.93	0.9	***0.93***	0.9	0.84	0.85	0.84	0.89	0.87	***0.87***
	EGPR	0.99	0.98	0.99	0.99	0.99	0.99	***0.99***	0.88	0.83	0.84	0.84	0.91	0.86	***0.86***
	MGPR	0.94	0.97	0.98	0.96	0.99	0.98	***0.97***	0.88	0.82	0.83	0.83	0.89	0.86	***0.85***
	LSVM	0.93	0.88	0.89	0.90	0.87	0.86	***0.89***	0.87	0.82	0.82	0.83	0.88	0.84	***0.84***
	FT	0.95	0.93	0.93	0.94	0.94	0.92	***0.94***	0.81	0.73	0.72	0.75	0.84	0.76	***0.77***
	LR	0.93	0.88	0.89	0.9	0.87	0.87	***0.89***	0.88	0.82	0.82	0.82	0.85	0.83	***0.84***
**MDWD to MDD**	***BT***	0.97	0.96	0.96	0.96	0.96	0.94	***0.96***	0.93	0.91	0.91	0.88	0.9	0.9	***0.91***
	EGPR	1.00	1.00	1.00	1.00	1.00	1.00	***1.00***	0.93	0.89	0.9	0.88	0.91	0.9	***0.90***
	MGPR	0.99	0.99	0.99	0.99	0.99	0.99	***0.99***	0.92	0.88	0.9	0.87	0.91	0.9	***0.90***
	LSVM	0.96	0.93	0.94	0.94	0.93	0.89	***0.93***	0.93	0.89	0.9	0.87	0.91	0.9	***0.90***
	FT	0.98	0.97	0.97	0.97	0.97	0.96	***0.97***	0.88	0.84	0.84	0.84	0.87	0.84	***0.85***
	LR	0.96	0.95	0.95	0.94	0.94	0.91	***0.94***	0.93	0.89	0.9	0.86	0.89	0.88	***0.89***

Table S2. *RMSE* of the ML models for the approaches *i) MDD to MDD ii) MWD to MDD & iii) MDWD to MDD*

Approach	Target Stations / ML Model	Training							Testing						
		Sylhet	Srimangal	Rangpur	Mymensingh	Dinajpur	Bogra	***Mean***	Sylhet	Srimangal	Rangpur	Mymensingh	Dinajpur	Bogra	***Mean***
**MDD to MDD**	***BT***	1.54	1.38	1.58	1.53	2.08	2.05	***1.69***	1.98	1.88	1.96	2.17	3.09	2.72	***2.30***
	EGPR	0.01	0.01	0.86	0.01	0.01	0.01	***0.15***	2.05	1.83	1.78	1.99	2.69	2.48	***2.14***
	MGPR	0.16	1.18	0.99	0.08	0.9	0.01	***0.55***	2.15	1.87	2	2.17	2.69	2.64	***2.25***
	LSVM	1.85	1.77	1.7	1.95	2.76	2.82	***2.14***	1.78	1.9	1.8	2.11	2.86	2.53	***2.16***
	FT	1.26	1.21	1.56	1.24	1.77	1.78	***1.47***	3.06	2.19	2.65	2.82	3.51	3.16	***2.90***
	LR	1.82	1.69	1.69	1.91	2.69	2.72	***2.09***	1.85	1.77	1.83	2.13	2.96	2.41	***2.16***
**MWD to MDD**	***BT***	1.82	1.86	1.89	1.85	2.61	2.28	***2.05***	2.21	2.39	2.32	2.55	3.22	2.77	***2.58***
	EGPR	0.72	0.83	0.69	0.73	0.66	0.65	***0.71***	2.49	2.42	2.39	2.54	3.15	2.81	***2.63***
	MGPR	1.82	1.14	0.99	1.38	1.03	1.13	***1.25***	2.49	2.49	2.44	2.62	3.19	2.83	***2.68***
	LSVM	2.01	2.23	2.15	2.12	3.39	2.86	***2.46***	2.61	2.57	2.56	2.66	3.52	3.01	***2.82***
	FT	1.66	1.73	1.71	1.64	2.31	2.13	***1.86***	3.11	3.15	3.22	3.3	3.86	3.84	***3.41***
	LR	1.97	2.16	2.11	2.07	3.28	2.7	***2.38***	3.53	2.48	2.52	2.68	3.71	3.13	***3.01***
**MDWD to MDD**	***BT***	1.35	1.34	1.29	1.41	1.89	1.79	***1.51***	1.93	1.81	1.83	2.18	2.99	2.44	***2.20***
	EGPR	0.01	0.01	0.01	0.01	0.01	0.01	***0.01***	1.97	1.99	1.88	2.18	2.76	2.34	***2.19***
	MGPR	0.39	0.31	0.14	0.21	0.37	0.21	***0.27***	2.08	2.05	1.94	2.26	2.82	2.42	***2.26***
	LSVM	1.48	1.63	1.48	1.66	2.44	2.39	***1.85***	1.86	1.99	1.87	2.25	2.89	2.39	***2.21***
	FT	1.16	1.13	1.04	1.18	1.68	1.54	***1.29***	2.54	2.46	2.55	2.6	3.49	3.11	***2.79***
	LR	1.39	1.48	1.38	1.57	2.29	2.22	***1.72***	1.91	1.93	1.94	2.36	3.2	2.59	***2.32***

Table S3. *R^2 & RMSE* of the ML models for the approaches *i) MDD to MDD ii) MWD to MDD & iii) MDWD to MDD* using testing dataset

Approach	*Target Stations* / *ML Model*	R^2 Sylhet	R^2 Srimangal	R^2 Rangpur	R^2 Mymensingh	R^2 Dinajpur	R^2 Bogra	R^2 ***Mean***	RMSE Sylhet	RMSE Srimangal	RMSE Rangpur	RMSE Mymensingh	RMSE Dinajpur	RMSE Bogra	RMSE ***Mean***
**MDD to MDD**	***BT***	0.81	0.76	0.78	0.73	0.79	0.76	***0.77***	3.15	3.04	3.01	3.33	4.11	4.65	***3.55***
	EGPR	0.80	0.76	0.82	0.72	0.79	0.71	***0.77***	3.37	3.04	2.72	3.43	4.14	3.98	***3.45***
	MGPR	0.75	0.74	0.80	0.69	0.80	0.68	***0.74***	3.7	3.18	2.85	3.55	4.13	4.15	***3.59***
	LSVM	0.79	0.74	0.80	0.71	0.77	0.73	***0.76***	3.36	3.23	2.99	3.56	4.89	4.18	***3.70***
	FT	0.58	0.72	0.70	0.62	0.70	0.70	***0.67***	4.96	3.38	3.64	4.16	5.1	4.13	***4.23***
	LR	0.80	0.74	0.78	0.69	0.78	0.72	***0.75***	3.35	3.19	3.15	3.63	4.78	4.12	***3.70***
**MWD to MDD**	***BT***	0.69	0.69	0.72	0.67	0.78	0.76	***0.72***	4.13	3.46	3.4	3.64	4.27	3.57	***3.75***
	EGPR	0.74	0.71	0.73	0.65	0.76	0.77	***0.73***	3.68	3.34	3.31	3.81	4.44	3.56	***3.69***
	MGPR	0.74	0.71	0.72	0.63	0.75	0.76	***0.72***	3.71	3.37	3.40	3.88	4.56	3.63	***3.76***
	LSVM	0.66	0.65	0.69	0.62	0.71	0.69	***0.67***	4.29	3.81	3.59	4.05	4.95	4.2	***4.15***
	FT	0.62	0.68	0.70	0.6	0.72	0.58	***0.65***	4.97	3.67	3.69	4.12	4.99	4.88	***4.39***
	LR	0.69	0.63	0.68	0.61	0.71	0.69	***0.67***	4.13	3.84	3.63	4.00	4.96	4.07	***4.11***
**MDWD to MDD**	***BT***	0.80	0.81	0.79	0.70	0.80	0.76	***0.78***	3.20	2.95	2.76	3.52	4.14	3.60	***3.36***
	EGPR	0.81	0.79	0.82	0.69	0.79	0.78	***0.78***	3.21	2.77	2.86	3.53	4.14	3.42	***3.32***
	MGPR	0.79	0.77	0.80	0.67	0.78	0.77	***0.76***	3.32	2.90	2.99	3.65	4.27	3.52	***3.44***
	LSVM	0.80	0.71	0.82	0.68	0.78	0.76	***0.76***	3.32	2.75	3.39	3.69	4.38	3.66	***3.53***
	FT	0.64	0.65	0.68	0.53	0.74	0.67	***0.65***	4.44	3.98	3.78	4.83	5.32	4.56	***4.49***
	LR	0.79	0.71	0.78	0.61	0.74	0.70	***0.72***	3.61	3.07	3.49	4.18	4.83	4.23	***3.90***
